# A historical overview of the need for distinction between the attainment of thermodynamic equilibrium and a kinetic steady state in experimental studies

**DOI:** 10.1007/s00249-026-01847-2

**Published:** 2026-07-22

**Authors:** Donald J. Winzor, David J. Scott

**Affiliations:** 1 School of Chemistry and Molecular Biosciences, University of Queensland, Brisbane, QLD 4072 Australia; 2 School of Biosciences, University of Nottingham, Sutton Bonington Campus, Sutton Bonington, LE12 5RD Loughborough, UK; 3 Research Complex at Harwell, Rutherford Appleton Laboratory, Oxfordshire, OX11 OFA Chilton, UK

**Keywords:** Ultracentrifugation, Electrophoresis, Chromatography, Nonspecific binding, Static light scattering

## Abstract

In the quantitative characterization of experimental systems the observation of a time-independent response can signify the attainment of thermodynamic equilibrium. However, there are also situations where the time-independence of experimental response merely reflects the attainment of a kinetic steady state. Although quantitative expressions derived on the basis of kinetic steady-state attainment retain validity for a system at thermodynamic equilibrium, those based on attainment of thermodynamic equilibrium do not apply to systems merely reflecting the attainment of a kinetic steady state. This survey of experimental procedures encountered during research endeavours over the past six decades has revealed many situations where the source of the time-independent instrumental response was correctly identified, but also a number where the responses were misinterpreted.

## Introduction

The present review emphasizes the importance of establishing whether the observation of a seemingly time-independent response in a variety of experimental situations reflects the attainment of thermodynamic equilibrium or merely a kinetic steady state. This dilemma arises in part from the impracticality of employing the theoretical definition of thermodynamic equilibrium as the state of an experimental system at infinite time ‒ an impasse that necessitates recourse to observation of time-independence of the signal being monitored as an acceptable experimental criterion for the essential attainment of thermodynamic equilibrium. However, that condition is also the criterion for attainment of a kinetic steady state.

The scientific literature contains many reports of situations where the observation of a steady state has been misinterpreted as signifying the attainment of thermodynamic equilibrium. In that regard a classic example of this mistake occurred in quantitative characterization of the rectangular hyperbolic dependence of initial velocity upon substrate concentration in enzyme kinetic studies (Michaelis and Menten 1913; Briggs and Haldane 1925). On the other hand, the concentration distributions observed in sedimentation equilibrium experiments afford situations where the steady state does reflect the attainment of thermodynamic equilibrium. Consequently, the data may be accorded rigorous interpretation based on either steady state (Lamm 1929) or thermodynamic equilibrium (Goldberg 1953) attainment. Action should therefore be taken to ascertain which situation applies to an observed time-independent experimental response before quantitative interpretation is attempted.

Here we draw attention to a diversity of experimental procedures in which the need for distinguishing between the attainment of a steady state and thermodynamic equilibrium has been encountered during our research efforts over the past six decades. The inclusion of studies involving techniques and approaches that no longer exist reflects the importance of their contributions to our current understanding of procedures that have survived the passage into the twenty first century.

## Enzyme kinetic studies: the Michaelis constant

The earliest biochemical example of the need for distinction between the attainment of a kinetic steady state and thermodynamic equilibrium was revealed in the context of enzyme catalysis (Michaelis and Menten 1913), for which interaction between enzyme (E) and substrate (S) requires interpretation in terms of the reaction scheme (Briggs and Haldane 1925)

1 $${\mathrm{E}} + {\mathrm{S}}\:\mathop \leftrightarrows_{{k_{2} }}^{{k_{1} }} \:{\mathrm{ES}}\:\mathop \to \limits^{{k_{3} }} \:{\mathrm{E}} + {\mathrm{P}}$$

In the original enzyme kinetic study (Michaelis and Menten 1913) the rectangular hyperbolic dependence of initial velocity (*v*) upon molar substrate concentration $\:{C}_{s}$ in an experiment with total enzyme concentration $\:{\stackrel{-}{C}}_{E}$was described by the expression

2 $$\:\frac{v}{V}=\frac{{C}_{ES}}{{\stackrel{-}{C}}_{E}}=\frac{{C}_{S}}{{(K}_{m}+{C}_{S})}\:$$

where $\:V={k}_{3}{\stackrel{-}{C}}_{E}$ denotes the maximum initial velocity and $\:{K}_{m}$ the Michaelis constant. Because the observation of a constant initial velocity was taken to signify the attainment of thermodynamic equilibrium between the enzyme−substrate complex and the two reactants, $\:{K}_{m}$ was identified with the dissociation constant $\:{K}_{d}={k}_{2}/{k}_{1}$. However, the more general interpretation of initial velocity in terms of steady-state attainment (Briggs and Haldane 1925) identifies the Michaelis constant as

3 $$\:{K}_{m}\:=\:\frac{({k}_{2}+{k}_{3})}{{k}_{1}}$$

which only becomes a reasonable approximation of its thermodynamic counterpart $\:{K}_{d}$ in situations where $\:{k}_{3}\ll\:{k}_{2}.$ The consequent concept of the Michaelis constant as a kinetic steady-state parameter is now well entrenched in enzymology doctrine.

## Analytical ultracentrifugation: sedimentation velocity of chemically inert systems

The development of an oil-turbine ultracentrifuge in the 1920s that could operate at rotor speeds as high as 60,000 rpm paved the way for the generation of forces up to 250,000 times that of gravity (Svedberg and Rinde 1924). Irrespective of the rotor speed selected, the migration of a noninteracting macromolecular solute (such as a protein) is described by the equation (Lamm 1929)

4 $$\:\left( {\:\frac{{\partial c}}{{\partial t}}} \right)_{r} = - \:\frac{1}{r}\:\frac{\partial }{r}\left\{ {r\left( {\omega ^{2} src - D\left( {\frac{{\partial c}}{{\partial r}}} \right)\:} \right)} \right\}$$

where *c* is the weight-concentration of solute at radial distance *r*, and *t* is the time of centrifugation at constant angular velocity *ω*. The sedimentation coefficient and translational diffusion coefficient of the solute are denoted by *s* and *D* respectively.

**Fig. 1:**
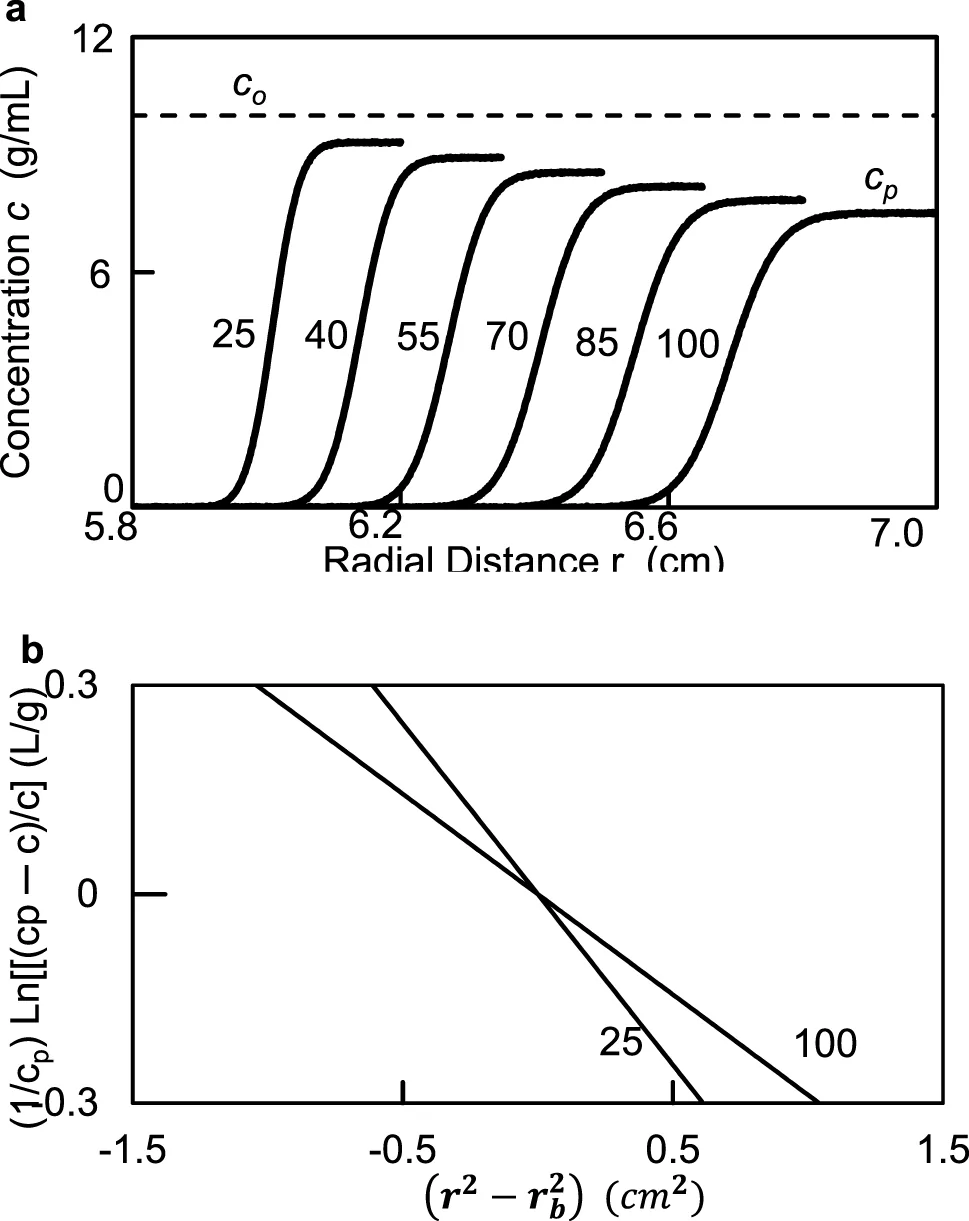
Predicted sedimentation velocity behaviour of a globular protein. (**a**) Simulated sedimentation velocity distributions for a 10 g/L horse γ-globulin solution after the indicated periods (min) of centrifugation at 56,100 rpm. (**b**) Use of Eq. (10) to demonstrate the lack of steady state attainment by virtue of the failure of analyses of the distributions to be described by a single dependence. [Data taken from Fig. 5 of Scott et al. 2015).]

Use of a high rotor speed leads to centrifugation of a sharp protein boundary from which the sedimentation coefficient is determined as

5 $$\:s = \:\frac{{dr_{p} /dt}}{{\omega ^{2} \mathop r\limits^{ - } }}\: = \:\frac{{\left( {d{\mathrm{ln}}\:r_{p} } \right)/dt}}{{\omega ^{2} }}$$

where $\:{r}_{p}$ denotes the radial position of the boundary at time *t*. However, the concentration gradient, $\:\left( {\:\frac{{\partial c}}{{\partial r}}} \right)_{t}$ thereby generated results in an opposing migratory force governed by the diffusion coefficient *D*, and hence the observation of a symmetrical boundary that spreads with time. This feature is illustrated in Fig. 1a by the time-dependence of simulated sedimentation velocity distributions (Scott et al. 2015) for horse γ-globulin (10 g/L) centrifuged at 56,100 rpm. In that regard the progressively decreasing plateau concentration $\:{c}_{p}$ reflects migration in a sector-shaped cell, for which the time-dependent decrease in plateau concentration is described by

6 $$\:({c}_{p}{)}_{t}=({c}_{p}{)}_{o}({r}_{a}/{r}_{p}{)}^{2}$$

where $\:({r}_{a}/{r}_{p})$ denotes the ratio of positions of the boundary at the commencement of centrifugation (the air−liquid meniscus $\:{r}_{a}$) to that after migration for time *t*. Such experiments are referred to as sedimentation velocity runs.

As is evident in Fig. 1a, the centrifugation of a non-interacting solute species at sufficiently high speed gives rise to a boundary that spreads with time (or distance migrated). However, that diffusional spreading is being countered to some extent by negative concentration dependence of the sedimentation coefficient.

From the Lamm expression for the radial-dependence of $\:(\frac{\partial c}{\partial t}{)}_{r}$, Eq. (4), it follows that attainment of a steady state, $\:(\frac{\partial c}{\partial t}{)}_{r}$= 0 for all *r*, would require conformity with the relationship

7 $$\omega ^{2} rc(s - s_{p} ) = D\:\frac{dc}{dr}$$

or on rearrangement,

8 $$\:\frac{1}{{rc}}\frac{{dc}}{{dr}}\: = \:\frac{{\omega ^{2} (s - s_{p} )}}{D}$$

where $\:{s}_{p}$ refers to the sedimentation coefficient in the plateau region with concentration $\:{c}_{p}$. Because the diffusion coefficient may be regarded as a constant over a moderate concentration range, conformity with the steady-state condition requires the variation in the left-hand side of Eq. (8) to be countered by the variation in sedimentation coefficient. The description of that *s−c* dependence varies with the nature of the macromolecular solute being investigated: a linear dependence of $\:{s}_{p}$ upon $\:{c}_{p}$ for compact globular proteins but a linear dependence of $\:1/{s}_{p}$ upon $\:{c}_{p}\:$for flexible macromolecules such as mucins as well as highly asymmetric proteins such as fibrinogen. The question at issue is whether the extent of boundary sharpening can *ever* counter the diffusional spreading to generate a hydrodynamic steady state.

### Globular proteins

For globular proteins the linear concentration dependence of the sedimentation coefficient is usually expressed in terms of the plateau concentration $\:{c}_{p}$ in the form

9 $$\:s={s}^{0}(1-{k}_{s}{c}_{p})$$

where $\:{s}^{0}$ is the limiting value of *s* as $\:{c}_{p}$ → 0, and $\:{k}_{s}$ is the concentration coefficient. Incorporation of the consequent expression for $\:(s-{s}_{p})$ into Eq. (8) followed by integration and subsequent rearrangement then gives rise to the expression (Creeth 1963,1964; Dishon et al. 1987)

10 $$\frac{1}{{c_{p} }}\mathrm{ln}[(c_{p} - c)/c] = \frac{{(\omega ^{2} s^{0} k_{s} )}}{{2D}}(r^{2} - r_{p}^{2} )$$

where $\:{r}_{p}$denotes the boundary position (radial distance at wich $\:c={c}_{p}/2)$ for the symmetrical boundaries obtained with globular proteins. Conformity with steady-state attainment requires coincidence of the dependencies of $\:(1/{c}_{p})\mathrm{ln}[({c}_{p}-c)/c]$ upon$\:({r}^{2}-{r}_{p}^{2})$ for all sedimentation distributions in which a plateau region is preserved. From Fig. 1b, which presents analysis of the simulated distributions (Fig. 1a) for horse γ-globulin ($\:{s}^{0}$ = 7.38 S, $\:{k}_{s}$ = 0.00785 L/g), and D = 4.8 ⋅ 10^‒7^ cm^2^/s), the systematic time dependence of the slope signifies that the extent of boundary sharpening arising from the negative s‒*c* dependence only partially counters the diffusional spreading of the boundary. Advantage has been taken of this partial boundary sharpening to devise procedures for determination of the diffusion coefficient from the time dependence of boundary spreading for a globular protein (Fujita 1956,1959; Baldwin 1957; Van Holde 1960; Winzor and Scott 2018). However, as noted by Creeth (1963,1964), an approximate steady state can be generated by speed manipulation during the velocity run. Its description as an approximate steady-state condition reflects the fact that $\:{c}_{p}$ varies with the duration of centrifugation (Fig. 1a).

A hydrodynamic steady state can also be generated in moving boundary electrophoresis (Hoch 1950), where boundary sharpening in the ascending limb of the U-tube assembly (Tiselius 1937) reflects a decreasing electrophoretic mobility across the migrating boundary. Although detailed discussion of theoretical aspects of the electrophoretic technique has been rendered irrelevant by the demise of moving boundary electrophoresis, it deserves mention as the stimulus for the corresponding sedimentation velocity studies (Creeth 1963,1964).

### Flexible macromolecular solutes

For relatively unstructured macromolecular solutes such as mucins the sedimentation coefficient is described by the relationship

11 $$\:s=\:\frac{{s}^{0}}{1+{K}_{s}{c}_{p}}$$

which is usually recognized from a linear dependence of * 1/s* upon $\:{c}_{p}$. Sedimentation coefficients for rigid rod-like proteins such as fibrinogen also conform with Eq. (11). Results continue to be described by Eq. (8) but with the difference in sedimentation coefficients given by

12 $$\:s-{s}_{p}=\:\frac{{s}^{0}{K}_{s}(c-{c}_{p})}{\left(1+{K}_{s}c\right)(1+{K}_{s}{c}_{p})}$$

which on combination with Eq. (8) and integration leads to the expression (Creeth 1963,1964)

13a $$\:\frac{{Y(1 + K_{s} c_{p} )}}{{K_{s} c_{p} }}\: = \:\frac{{\omega ^{2} s^{0} }}{{2D}}(r^{2} - r_{p}^{2} )$$

13b $$Y=\:\mathrm{l}\mathrm{n}\left(\frac{c}{{c}_{o}}\right)-\left(1+{K}_{s}{c}_{p}\right)-\left(1+{K}_{s}{c}_{p}\right)\mathrm{l}\mathrm{n}\left(\frac{{c}_{p}-c}{{c}_{o}}\right)$$

**Fig. 2:**
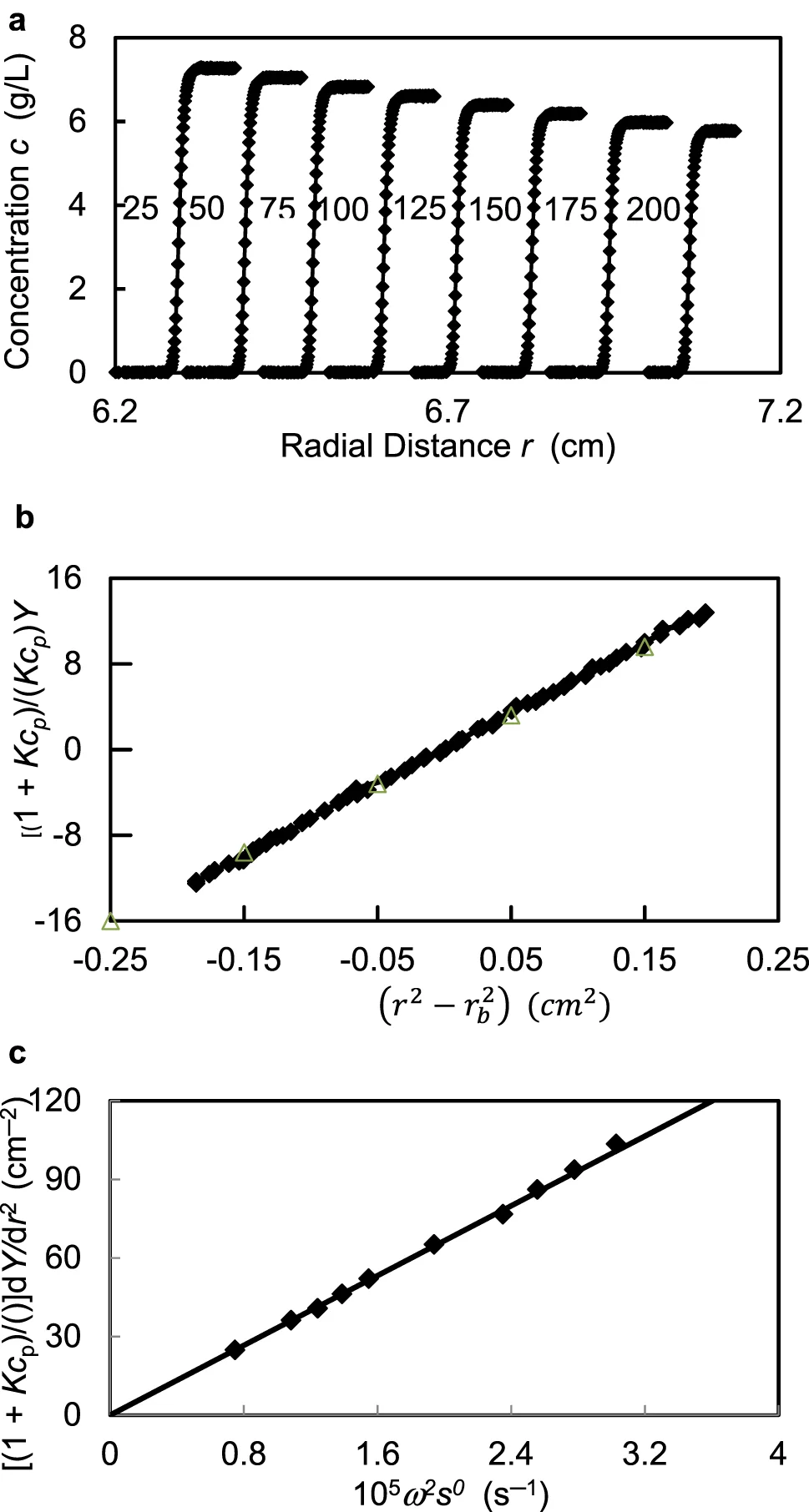
Simulated migration of a 7.5 g/L solution of a mucin preparation ($\:{s}^{o}$ = 11.1 S, $\:{K}_{s}$ = 0.12 L/g, *D* = 1.5 ⋅ $\:{10}^{-7}$ cm^2^/s (Scott et al. 2015) subjected to centrifugation in an experiment with the air liquid meniscus $\:{r}_{a}$ located at 6.2 cm. (**a**) Concentration distributions obtained after centrifugation at 40,000 rpm for the indicated times (min). (**b**) Conformity of these simulated distributions with Eq. (8). (**c**) Test for compliance of the slopes obtained from analyses of simulated runs at speeds in the range 25,000−50,000 rpm with steady-state attainment: the solid line denotes the theoretical slope predicted by Eqs. (13a, b). [Data taken from Figs. 1 and 4 of Scott et al. 2015).]

For these systems the location of the boundary position $$\:{r}_{p}$$is not straightforward because of boundary asymmetry. However, in the present context it can also be regarded (Creeth 1964) as the radius at which $$\:\mathrm{ln}c=(1+K{c}_{p})\mathrm{ln}\left({c}_{p}-c\right)$$ because an estimate of the diffusion coefficient *D* is already available from the slope of the dependence of $$\:[\left(1+{K}_{s}{c}_{p}\right)\:\mathrm{l}\mathrm{n}({c}_{p}-c)-\mathrm{ln}\mathrm{c}$$] upon $$\:{r}^{2}$$ in situations where values of $$\:{s}^{0}$$ and $$\:{K}_{s}$$ have been pre-determined. It then suffices to regard $$\:{r}_{p}$$ as the radial distance at which *Y* = 0. In the original applications of these quantitative expressions (Creeth 1963,1964) the centrifugation−diffusion‒ centrifugation regime used for horse γ-globulin was also adopted in the study of a mucin. However, subsequent numerical simulations (Dishon et al. 1987; Scott et al. 2015) have signified that the greater * s-c* dependence exhibited by this less structured macromolecule should give rise to spontaneous generation of an approximate steady state. In Fig. 2a advantage has been taken of the SEDFIT program (Schuck 1998, 2000) to simulate the time dependence of sedimentation velocity distributions for a 7.5 g/L solution of the mucin ($$\:{s}^{o}$$ = 11.1 S, $$\:{K}_{s}$$ = 0.12 L/g, * D* = 1.5 ⋅ $$\:{10}^{-7}$$$$\:{\mathrm{c}\mathrm{m}}^{2}/\mathrm{s})$$ centrifuged at 40,000 rpm (Scott et al. 2015). A striking feature of these distributions is the similarity of shape during migration across the entire cell length. The degree of conformity with attainment of the steady state condition is evident from Fig. 2b, which plots the data according to Eq. (13): data from four of the patterns in Fig. 2a (*t* = 25, 75, 125 and 200 min) are included. Considerable latitude in the choice of rotor speed compatible with steady state attainment is evident from Fig. 2c, which combines the slope of Fig. 2b with those of comparable pots derived from distributions in simulated runs with rotor speeds ranging from 20,000 to 50,000 rpm. A more critical factor governing the feasibility of steady state attainment is the magnitude of the product$$\:{K}_{s}$$$$\:{c}_{p}$$, which determines the extent of boundary sharpening. A decrease in the magnitude of $$\:{K}_{s}$$ and/or $$\:{c}_{p}$$. may well lead to the situation encountered in Fig. 1 with globular proteins, where the extent of sharpening ($$\:{K}_{s} \:{c}_{p}$$ in that case) only leads to a decreased extent of boundary spreading.

Evaluation of the diffusion coefficient from the slope, $\:{\omega}^{2}{s}^{0}/\left(2D\right)$, of Fig. 2b clearly affords a relatively simple analytical solution for its measurement by sedimentation velocity experiments on flexible macromolecular solutes exhibiting negative linear concentration dependence of 1/*s*. In that regard it should be noted that the steady state attained here differs from that being sought by Creeth (1963,1964), whose aim was to achieve the corresponding time-independence of a boundary exhibiting diffusional spreading. Indeed, a steady-state concentration distribution such as that shown in the current Fig. 2a after centrifugation for 25 min at 40,000 rpm would have emanated during the initial step. At that stage the rotor speed was decreased markedly to allow the regeneration of a boundary exhibiting a sufficient extent of boundary spreading to be compatible with transformation into a steady-state situation by further manipulation of the rotor speed. Although the same expressions apply to either form of steady state, greater precision of the diffusion coefficient determined from the slope of the plot of data according in accordance with Eq. 13, as in the current Fig. 2b, should emanate from the Creeth (1963,1964) approach because of an enhanced range of values for the abscissa parameter, ($\:{r}^{2}-{r}_{p}^{2})$. Unfortunately, the technical complexities associated with that procedure for achieving a steady state has led to its disregard.

In the current simpler approach, the steady state reflects situations where diffusional spreading is counterbalanced by the boundary sharpening effected by the *s*─*c* dependence. Closer inspection of the patterns in Fig. 2a reveals that those distributions reflect essentially a concentration discontinuity at the boundary position r_p_ wherein c is zero for *r* *<* r_p_ and c_p_. for r > r_p_. The potential for existence of a discontinuous *c versus r* distribution was pointed out by De Vault (1943) in a theoretical treatment of chromatography; and observed experimentally in a sedimentation velocity study of calf thymus [Fig. 1a of Cecil and Ogston (1948)], where the sedimentation coefficient was determined from the migration rate of a vertical spike in the concentration gradient distribution, *dc*/*dr versus r*. Such hypersharp (diffusion-free) distributions are also encountered in theoretical considerations of the mass-migration behaviour of solutes undergoing rapid, reversible association (Gilbert and Jenkins 1959; Nichol and Ogston 1965a).

Even though the technical complexities have now disappeared with the demonstration of spontaneous steady-state attainment in conventional sedimentation velocity experiments (Dishon et al. 1987; Scott et al. 2015), developments in computer technology have rendered possible the replacement of analytical approaches by numerical solution of nonlinear differential equations in ultracentrifugation using software packages such as SEDFIT (Schuck 2016), SEDANAL (Sherwood and Stafford 2016) and SEDNTERP (Philo 2023). Those technological developments have undermined to some extent the importance of the ingenuity displayed by earlier researchers in the field (Fujita 1956,1958; Baldwin 1957; Van Holde 1960; Creeth 1963,1964) at a time when analytical solution was the only option.

## Analytical ultracentrifugation: ideal sedimentation equilibrium of chemically inert systems

**Fig. 3:**
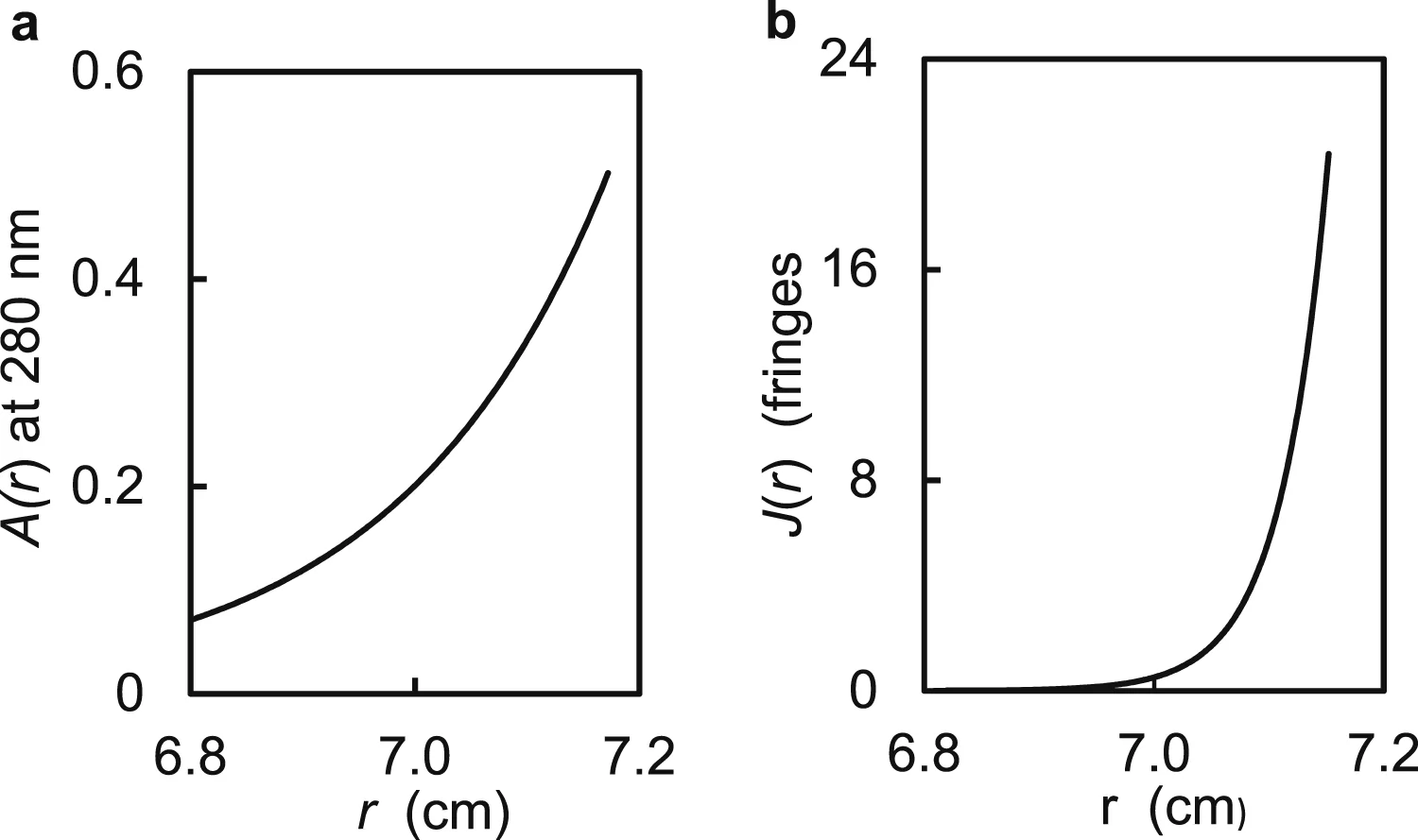
Sedimentation equilibrium distributions for carbonic anhydrase from experiments of low-speed (Van Holde and Baldwin 1958) and meniscus-depletion (Yphantis 1964) design. (**a**) Absorption optical record from a run at low-speed (15,000 rpm). (**b**) Rayleigh interferometric record of a run at 32,000 rpm, a rotor speed sufficiently high to ensure meniscus depletion and hence the measurement of protein concentration rather than $\:c\left(r\right)-c\left({r}_{a}\right)$. [Data taken from Fig. 1 of Winzor et al. (2001).]

The selection of a lower rotor speed clearly slows down the rate of centrifugal migration and hence decreases its dominance over the opposing diffusional process. Indeed, the continuation of centrifugation eventually results in a time-independent distribution [$\:(\frac{\partial c}{\partial t}{)}_{r}$= 0 for all *r*)]. Sedimentation equilibrium distributions of low-speed (Van Holde and Baldwin 1958) and meniscus-depletion Yphantis 1964) design for carbonic anhydrase (Winzor et al. 2001) are shown in Fig. 3a and b respectively.

### Treatment as a hydrodynamic steady state

Despite the designation of patterns such as Fig. 3a and b as sedimentation equilibrium distributions, the early use of Eq. (4) for the analysis of such concentration distributions merely presumes the attainment of a hydrodynamic steady state between sedimentation and diffusion processes. From Eq. (4) it follows that

14 $$\:{\omega}^{2}src=Ddc/dr$$

where the concentration gradient term replaces its partial counterpart in Eq. (4) because of redundancy of the time proviso. At that time very little advantage could be taken of this procedure for determining the magnitude of * s/D* and hence $\:{M}_{b},\:\:$the buoyant molecular mass of the macromolecular solute, by its incorporation into the Svedberg equation

15 $$M_{b} = \frac{{RTs}}{D} = \frac{{(RTdc/dr)}}{{(\omega ^{2} rc)}}$$

in which *T* is the absolute temperature and *R* the universal gas constant. This situation reflected the standard procedure of filling the cell with solute solution, a practice that necessitated centrifugation at constant angular velocity for three-to-four weeks and therefore placed an unacceptable demand on protein stability.

A potential breakthrough for the practical utilization of this steady-state expression occurred two decades later when Archibald (1947) pointed out that a condition analogous to a hydrodynamic steady-state (no flow and therefore no net solute flow) invariably applied at two positions in the liquid column being subjected to centrifugation − the air−liquid meniscus $\:{r}_{a}$ and the cell base $\:{r}_{b}$: However, considerable improvement to the optical system of the electrically driven Spinco (later Beckman) model E ultracentrifuge (Klainer and Kegeles 1955, 1956) was required for the advantage to be taken of this theoretical development.

**Fig. 4:**
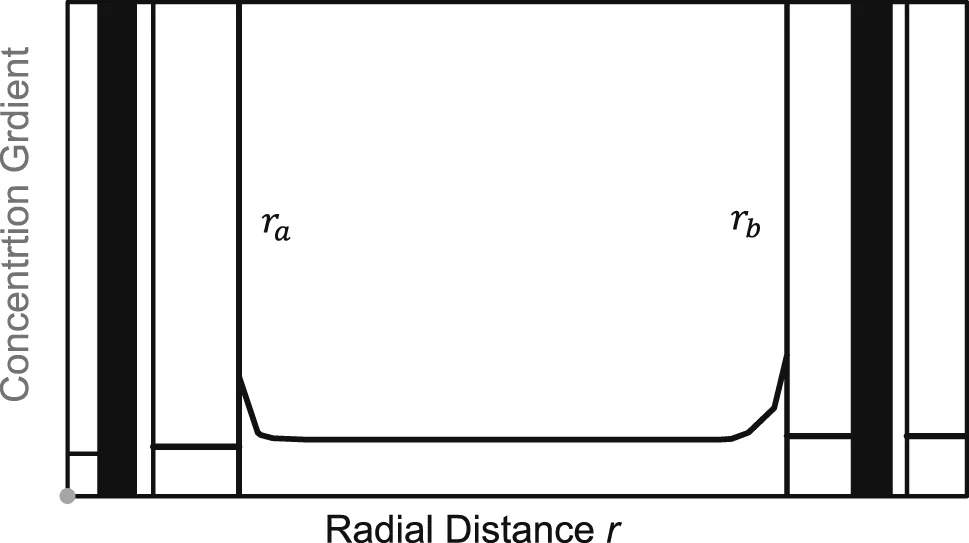
Schematic schlieren record of an approach-to-equilibrium sedimentation distribution during the early stages of centrifugation. The existence of a plateau region $\:(c={c}_{o},dc/dr=0)$ renders feasible the application of Eqs. (15), (16a) and (16b) to obtain the buoyant molecular mass of the protein being studied [Klainer and Kegeles (1955,1956).]

In those days ultracentrifuges were only fitted with a schlieren optical system that provided a sedimentation distribution in derivative form, $\:dc/dr\:~versus~ \:r,\:$which yielded the required values of $\:(dc/dr{)}_{a}$ and/or (*dc/dr*$\:{)}_{b}$; but the application of Eq. (15) to these liquid column extremities also requires knowledge of $\:{c}_{a}\:$and/or $\:{c}_{b}$. This requirement was met by employing approach-to-equilibrium optical records from early stages of centrifugation at angular velocity *ω*: the pattern then contained a plateau region (* dc/dr=0***)** where the concentration could be identified with the loading concentration $\:{c}_{o}$. This feature of an Archibald approach-to-equilibrium experiment is illustrated in Fig. 4, which also reflects the inclusion of a layer of carbon tetrachloride in the loading process to achieve better delineation of $\:{r}_{b}$, the lower radial limit of the protein solution. The solute concentrations at the two liquid column extremities ($\:{c}_{a}$, $\:{c}_{b}$) were then calculated as

16a $$\:{c}_{a}={c}_{0}-\frac{1}{{r}_{a}^{2}}\underset{{r}_{a}}{\overset{r}{\int\:}}{r}^{2}\left(dc/dr\right)dr$$

16b $$\:{c}_{b}={c}_{0}+\frac{1}{{r}_{b}^{2}}\underset{r}{\overset{{r}_{b}}{\int\:}}{r}^{2}\left(dc/dr\right)dr$$

    

using trapezoidal approximation for evaluation of the integrals and a synthetic boundary experiment on a sample of the loaded solution to obtain$\:{\:c}_{o}$.

### Consideration in terms of thermodynamic equilibrium

During those attempts to render technically feasible the Archibald procedure for molecular weight determination Goldberg (1953) recognized that the sedimentation equilibrium technique could be accorded full thermodynamic status by expressing the opposing (diffusional) force [* Ddc/dr* in Eqs. (7) and (14)] as $\:d\mu/dc$, the gradient in solute chemical potential. On the basis that the solute chemical potential for an ideal solution can be written in terms of its standard-state value,$\:\:{\mu}^{o},$ as $\:\mu={\mu}^{o}+RT\mathrm{l}\mathrm{n}c$, the differential equation describing the sedimentation equilibrium condition becomes

17 $$\:{M}_{b}{\omega}^{2}rdr=RTd\mathrm{l}\mathrm{n}c$$

with the buoyant molecular mass defined in terms of solute molecular mass *M*, its partial specific volume $\:\overline{v\:}$ and the solvent density $\:{\rho}_{s}$ as (Wills and Winzor 1992; Wills et al. 1993, 2001)

18 $$\:{M}_{b}=M(1-\overline{v}{\rho}_{s})$$

Integration of Eq. (17) with a reference radial distance $\:{r}_{F}\:$and *r* as limits gives rise to the expression

19 $$\:\frac{{M}_{b}{\omega}^{2}({r}^{2}-{r}_{F}^{2})}{2RT}=\mathrm{l}\mathrm{n}\left(\frac{c\left(r\right)}{c\left({r}_{F}\right)}\right)$$

which, for direct analysis of concentration distributions, is most readily expressed (Haschemeyer and Bowers 1970) as

20 $$\:c\left(r\right)=c\left({r}_{F}\right)\mathrm{exp}\left[\frac{{M}_{b}{\omega}^{2}({r}^{2}-{r}_{F}^{2})}{2RT}\right]$$

Nonlinear least-squares analysis of a set of [$\:r,\:c\left(r\right)]$ measurements obtained at constant temperature and angular velocity in terms of Eq. (20) thus yields estimates of the solute concentration at the reference radial position, $\:c\left({r}_{F}\right),$ and the buoyant molecular mass of the protein, *M*_*b*,_ as curve-fitting parameters (Haschemeyer and Bowers 1970).

The demonstration that steady-state distributions could be accorded full thermodynamic status (Goldberg 1953) was soon followed by removal of the earlier practical impediment to the conduct of sedimentation equilibrium studies − the unreasonably long time required to achieve such concentration distributions for a 9 mm liquid column. Because the time for effective attainment of sedimentation equilibrium is proportional to $\:({r}_{b}-{r}_{a}{)}^{2}$, a three- to four-fold decrease in that column length allows effective equilibrium to be attained within 24–48 h (Van Holde and Baldwin 1958). This development has led to the virtual demise of the use of steady-state expressions in the analytical approach to quantitative analysis of sedimentation equilibrium distributions; and to the incorporation of rigorous allowance for the consequences of thermodynamic nonideality (Wills and Winzor 2001); Wills et al. 1996, 2001, 2012) on the statistical-mechanical basis of excluded volume (McMillan and Mayer 1945).

## Studies of interactions between dissimilar reactants

Biology abounds with reacting species that coexist in rapid association equilibrium with complex(es) thereof ‒ situations that will be simplified for present illustrative purposes by considering the interaction between reactants A and B to form a single complex C governed by association equilibrium constant *K*:

21 $$\:A+B\rightleftharpoons C\:;\:K=\:\frac{{C}_{C}}{{C}_{A}{C}_{B}}$$

On the grounds that the constituent (total) molar concentrations $\:{\overline{C}}_{A}$ and $\overline{C}_{B}$ (Tiselius 1930; Alberty and Marvin 1950) are given by

22a $$\:{\overline{C}}_{A}={C}_{A}+K{C}_{A}{C}_{B}$$

22b $$\:{\overline{C}}_{B}={C}_{B}+K{C}_{A}{C}_{B}$$

It is readily shown that the concentrations of free reactants are related to their constituent counterparts in the reaction mixture by the quadratic expressions

23a $$\:K{C}_{A}^{2}+\left[1+K\left({\overline{C}}_{B}-{\overline{C}}_{A}\right)\right]{C}_{A}-{\overline{C}}_{A}=0$$

23b $$\:K{C}_{B}^{2}+\left[1+K\left({\stackrel{-}{C}}_{A}-{\stackrel{-}{C}}_{B}\right)\right]{C}_{B}-{\stackrel{-}{C}}_{B}\:=0$$

Inasmuch as the estimation of either $\:{C}_{A}$ or $\:{C}_{B}$ in a mixture with composition $\:{\overline{C}}_{A},\:{\overline{C}}_{B}$ is required for the evaluation of *K*, the simplest approach entails the design of any migration experiment involving solute separation in such a way that a region with the original mixture composition is maintained throughout the run. Migration methods that have been pivotal to an understanding of the distinction between the attainment of thermodynamic equilibrium and a kinetic steady state in heterogeneous associations are considered in turn.

### Moving boundary electrophoresis

Such a situation is maintained in moving boundary electrophoresis (Tiselius 1937), where proteins were characterized in terms of a migration rate (*dx/dt*) under the influence of an electric field (potential gradient) *E*. The normalized parameter,

24 $$\:u=\:\frac{dx/dt}{E}$$

was termed the electrophoretic mobility of the protein. Because moving boundary electrophoresis for the analysis of protein mixtures was relegated to historical status by the advent of gel electrophoresis (Smithies 1955; Raymond and Weintrub 1959), some basic tenets of the U-tube electrophoresis cell are outlined here: a more detailed account of the apparatus appears elsewhere (Winzor 2004).

**Fig. 5:**
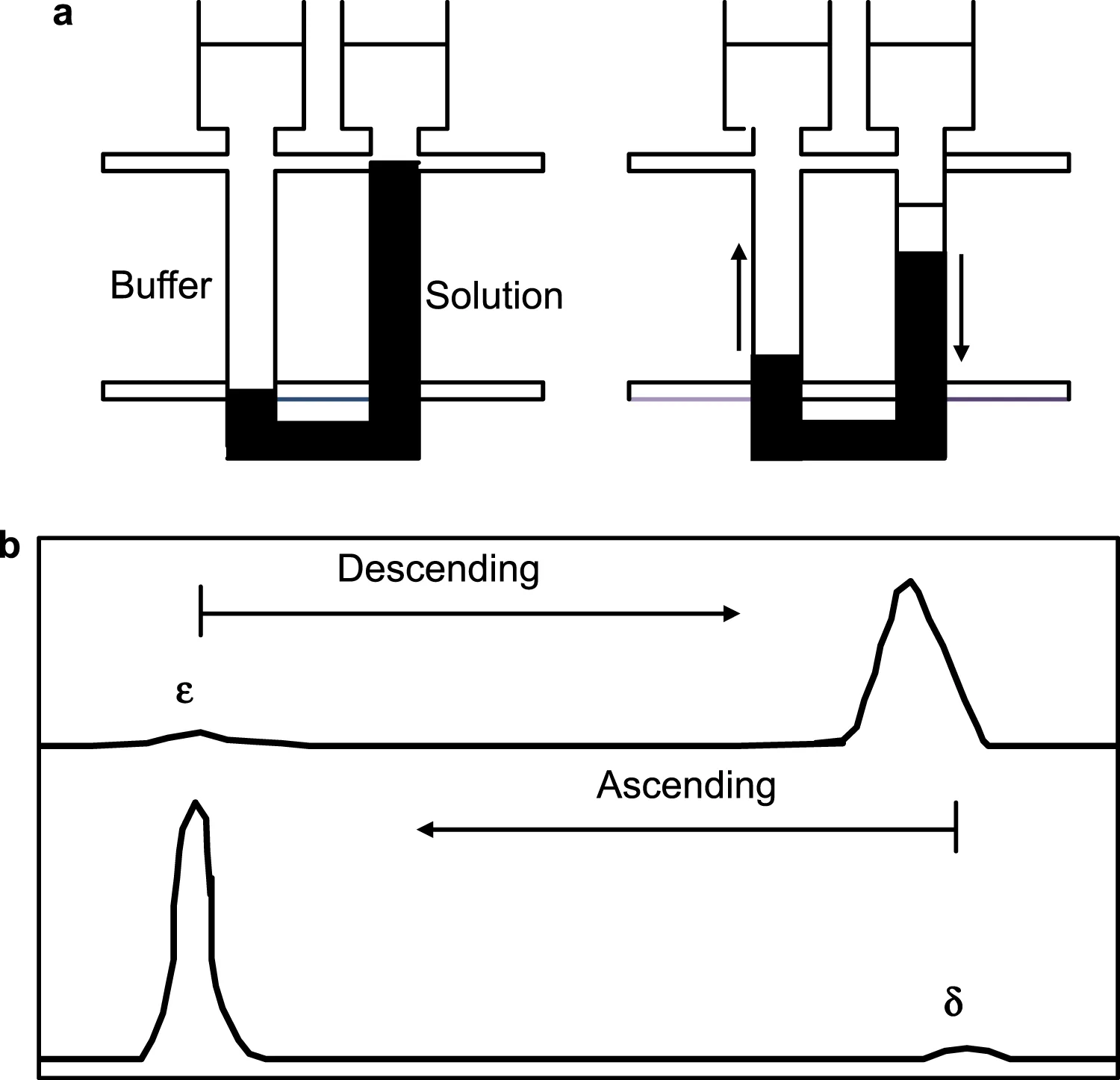
Moving boundary electrophoresis. (**a**) Schematic representation of the U-tube cell assembly. (**b**) An adaptation of schlieren patterns obtained by electrophoresis of bovine serum albumin in phosphate buffer, pH 8.6, *I* 0.1 M. [Data taken from Alberty (1948).]

As shown in Fig. 5a, the electrophoresis cell (rectangular in cross-section) was constructed in three sections with glass flanges that could be slid relative to each other to facilitate the formation of sharp boundaries. The right-hand centre and bottom sections contained protein solution, which was overlaid by diffusate against which the protein solution had been extensively dialyzed. Because the boundaries in the aligned cell were obscured by the ground-glass sliding flanges used for their creation, a small quantity of buffer was carefully removed from the left-hand side to bring the sharp boundaries into view (right-hand side of Fig. 5a). The application of an electric field in the appropriate direction then caused the boundary in the left-hand side to rise (ascend), whereas the other boundary descended.

An alternative form of the schlieren patterns ($\:dc/dx~\mathrm{v}\mathrm{e}\mathrm{r}\mathrm{s}\mathrm{u}\mathrm{s}\:~x$) obtained (Alberty 1948) for bovine serum albumin in phosphate buffer (pH 8.6, I 0,1 M) is shown in Fig. 5b.

Moving boundary electrophoresis was the first mass migration technique to find favour for characterizing mixtures of dissimilar macromolecular reactants coexisting in rapid association equilibrium with 1:1 complex (Longsworth 1959). Its provision of ascending and descending boundary patterns placed the method at a decided advantage over sedimentation velocity, which only yields the counterpart of the descending profile. Whereas the descending boundary monitors the movement of the reaction mixture species within a region already occupied by the equilibrium reaction mixture, the ascending boundary system records the migration of an initially sharp boundary into a region occupied by buffer.

**Fig. 6:**
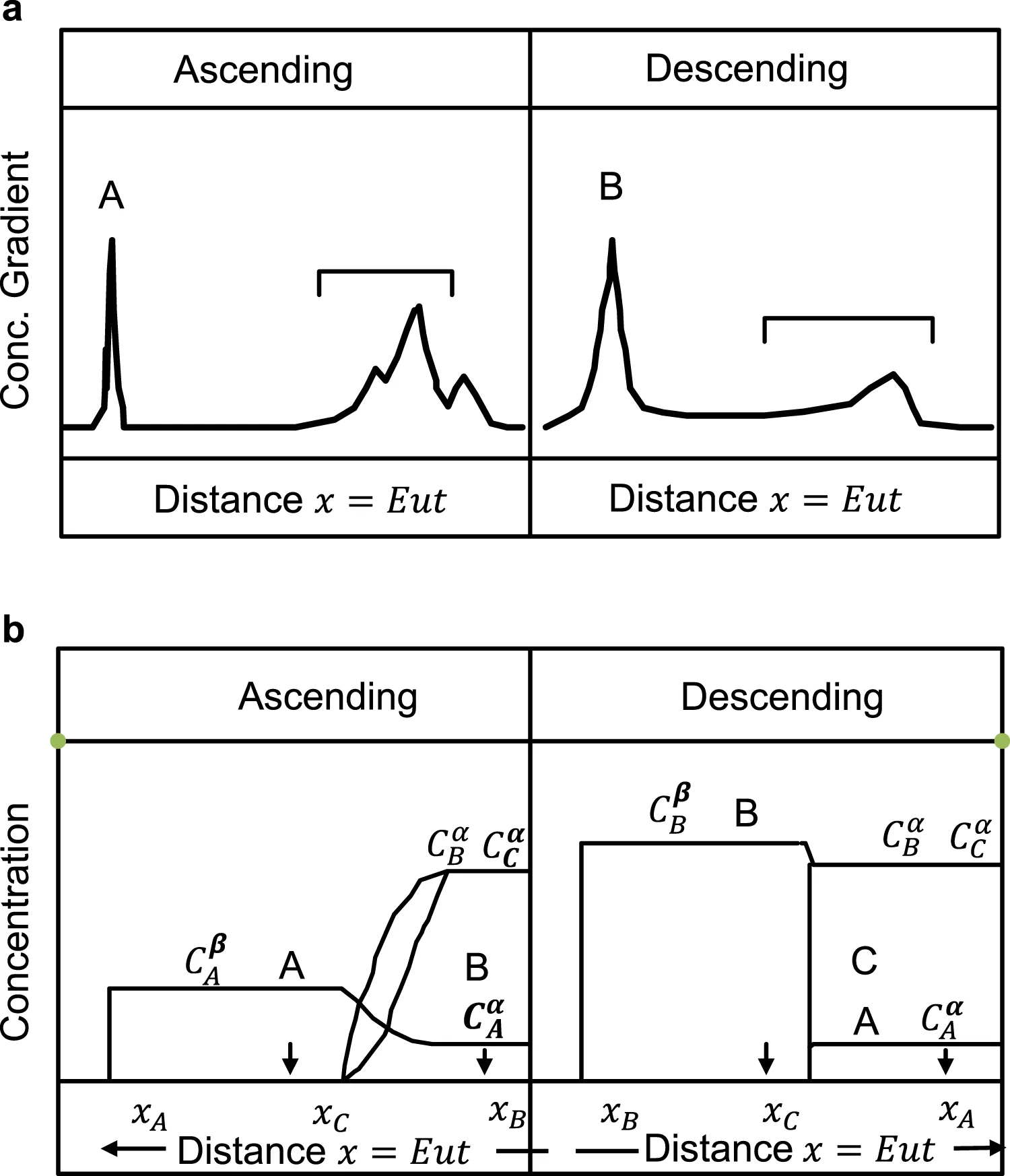
Moving boundary electrophoresis of a rapidly interacting system A + B *⇆* C with $\:{u}_{\mathrm{A}}>{u}_{C}>{u}_{B}.\:$ (**a**) Ascending and descending patterns obtained for a mixture of 5.5 g/L ovomucoid (B) and 8.7 g/L yeast nucleic acid (A) in acetate buffer, pH 4.63: reaction boundaries are denoted by square brackets. [Data taken from Longsworth (1959).] (**b**) Theoretical asymptotic patterns for this combination of mobilities [Data taken from Fig. 2 of Gilbert and Jenkins (1959),]

A reaction mixture in rapid association equilibrium is then recognized immediately by the non-enantiography (different shapes) of the two boundary patterns ‒ a feature evident from Fig. 6a that was obtained (Longsworth 1959) for a mixture of ovomucoid and yeast nucleic acid. Whereas the ascending pattern comprises a boundary of faster-migrating reactant A (nucleic acid) and a reaction boundary (bracketed), the free-solute boundary in the descending pattern reflects the slower-migrating reactant B (ovomucoid). Similar findings were observed in moving boundary electrophoresis of antibody‒antigen (Pepe and Singer 1959) and pepsin‒serum albumin (Cann and Klapper 1961) reaction mixtures. As realized in those experimental investigations and confirmed by systematic theoretical studies (Glibert and Jenkins 1959; Nichol and Ogston 1965a), such behaviour characterizes electrophoretic patterns for reaction mixtures because the dependence of mobility on the charge/size ratio ensures an intermediate migration rate for complex $\:({u}_{A}>{u}_{C}>{u}_{B})$. The predicted electrophoretic behaviour (Gilbert and Jenkins 1959) for this velocity combination in a reacting mixture with equal molar concentrations of complex (C) and slow reactant (B) is shown in Fig. 6b, where the original composition is designated as $\:[{C}_{A}^{\alpha},\:{C}_{B}^{\alpha},\:{C}_{C}^{\alpha}]$.

An important feature to emerge from Fig. 6b is the failure of the concentration across either boundary of pure reactant boundary ($\:{C}_{A}^{b},\:{C}_{B}^{\beta}$) to provide a direct measure of its concentration $\:({C}_{A}^{\alpha}\:,\:{C}_{B}^{\alpha}$) in the equilibrium mixture subjected to electrophoresis. $\:\:{C}_{A}^{b}~\mathrm{a}\mathrm{n}\mathrm{d}~\:{C}_{B}^{\beta}$ must therefore be regarded as steady-state (not equilibrium) parameters. As realized in those early experimental and theoretical studies, information about their magnitudes for a 1:1 interaction can be obtained by introducing the concepts of constituent concentrations and constituent velocities (Tiselius 1930; Alberty and Marvin 1950), defined by

25a $$\:{\overline{C}}_{A}^{\alpha}={C}_{A}^{\alpha}+{C}_{C}^{\alpha}\:\:\:\:\:\:\:\:\:\:{\overline{u}}_{A}=\:\frac{{C}_{A}^{\alpha}{u}_{A}+{C}_{C}^{\alpha}{u}_{C}}{{\overline{C}}_{A}^{\alpha}}$$

25b $$\:{\overline{C}}_{B}^{\alpha}\:={C}_{B}^{\alpha}+{C}_{C}^{\alpha}\:{\overline{u}}_{B}=\:\frac{{C}_{B}^{\alpha}{u}_{B}+{C}_{C}^{\alpha}{u}_{C}}{{\overline{C}}_{B}^{\alpha}}$$

    

Because the B constituent exists in some form on both sides of the descending αβ reaction boundary in Fig. 6b, a continuity equation expressing mass balance may be written (Longsworth 1959; Nichol and Ogston 1965a) as

26 $$\:{{\overline{u}}_{B}{\overline{C}}_{B}^{\alpha}-{u}_{B}C}_{B}^{\alpha}={\overline{u}}_{A}({\overline{C}}_{B}^{\alpha}-{C}_{B}^{\alpha})$$

After substitution of expressions from Eqs. (25a) and (25b) for the constituent quantities, Eq. (27) can be rearranged (Longsworth 1959) to allow delineation of the equilibrium concentration of faster-migrating reactant as

27 $$\:{C}_{A}^{\alpha}=({\overline{C}}_{A}^{\alpha}+{C}_{B}^{\beta}-{\overline{C}}_{B}^{\alpha})\:\frac{{\overline{u}}_{A}-{u}_{B}}{{u}_{A}-{u}_{B}}$$

and hence determination of the association equilibrium constant *K* from the expression

28 $$\:K=\:\frac{{C}_{C}^{\alpha}}{{C}_{A}^{\alpha}{C}_{B}^{}}\:=\:\frac{{\overline{C}}_{A}^{\alpha}-{C}_{A}^{\alpha}}{{C}_{A}^{\alpha}({\overline{C}}_{B}^{\alpha}-{\overline{C}}_{A}^{\alpha}+{C}_{A}^{\alpha})}$$

More stringent consideration of the mass conservation aspect (Nichol and Ogston 1965b) has shown that Eq. (28) should be written as

29 $$\:{C}_{A}^{\alpha}\:=\:\frac{{\overline{C}}_{A}^{\alpha}({\overline{u}}_{A}-{u}_{B})+\left({C}_{B}^{\beta}-{\overline{C}}_{B}^{\alpha}\right)({u}_{B}^{{\prime\:}}-{u}_{B})}{{u}_{A}-{u}_{B}}$$

The assumption inherent in Eq. (28) is that the median bisector of the combined A and B constituent gradients across the αβ boundary provides an acceptable approximate estimate of $\:{\overline{u}}_{A}$ and $\:{u}_{B}^{{\prime\:}}$, where $\:{u}_{B}^{{\prime\:}}$ reflects the median bisector of the gradient in B constituent across the αβ boundary in the descending pattern in Fig. 6b, and $\:{\overline{u}}_{A}$ the corresponding parameter for the A constituent. Although the assumed identity of these two parameters is not necessarily valid, there seems to be no alternative to this approximation in experiments with refractometric recording of the summed gradients in the two constituents.

In summary, the concentrations of pure-solute boundaries observed in moving boundary electrophoresis of interacting mixtures are clearly not equilibrium parameters. However, no confusion has arisen because their identity as steady-state parameters was recognized from the outset.

### Frontal analysis continuous electrophoresis

**Fig. 7:**
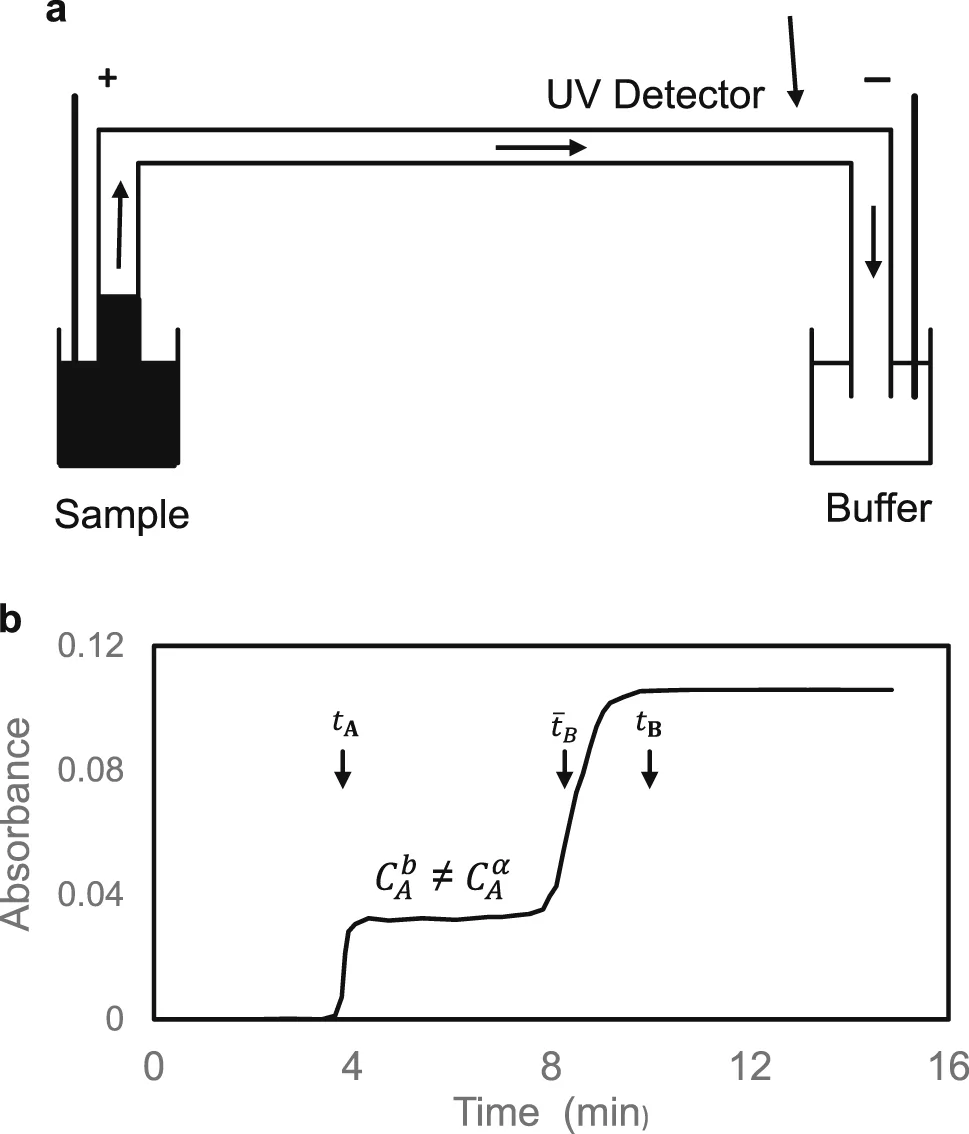
Frontal analysis continuous capillary electrophoresis. (**a**) Schematic representation of the set-up at the start of electrophoresis. (**b**) Pattern obtained for a mixture of 1.0 g/L β-lactoglobulin and 0.2 g/L sodium poly(styrenesulphonate) in 0.05 M phosphate buffer, pH 6.7. Vertical arrows denote the retention times (*t*_*A*_, *t*_*B*_) of the two reactants as well as the constituent retention time of the slower-migrating reactant. [Data taken from Fig. 2 of Gao et al. (1997).]

Frontal analysis continuous capillary electrophoresis (Gao et al. 1997, 1998; Hattori et al. 2000) is a technique developed much later to generate the counterpart of the ascending pattern in moving boundary electrophoresis. One end of a capillary filled with buffer is inserted into a vial of interacting mixture, whereas the vial at the other end contains more of the buffer in the capillary. The two vials also serve as electrode vessels (Fig. 7a). Selection of the left-hand electrode as the anode for a mixture in which reactants and complexes all bear net negative charge signifies the dominance of electroosmotic flow, which is opposed by electrophoretic migration: the species with smallest electrophoretic migration rate thus becomes the fast-moving species (A). The generated migrating boundary pattern is monitored as a time-dependence of absorbance at a fixed point 20 cm from the capillary inlet (Fig. 7a).

The technique has been used to characterize the reversible interactions of protein ligands (β-lactoglobulin and bovine serum albumin) with sodium poly(styrenesulphonate) by considering the size of the faster-migrating boundary, $\:{C}_{A}^{b}$, to reflect the concentration of free protein in the equilibrium mixture subjected to electrophoresis. From Eq. (29) such action presumes the comigration of the polymer (A) and polymer−protein complex (C). The validity of that assumption has been called into question (Winzor 2006) because of the electrophoresis pattern (Fig. 7b) for a mixture of β-lactoglobilin and sodium poly(styrenesulphonate) in 0.05 M phosphate buffer, pH 6.7 (Gao et al. 1998). Also shown are the respective retention times $\:{t}_{B}$ and $\:{t}_{A}$ of the polymeric acceptor (B) and protein ligand (A), as well as that ($\:{\overline{t}}_{B}$) for the reaction boundary across which the total polymer concentration rises from zero to its equilibrium value ($\:{C}_{B}^{\alpha}+{C}_{AB}^{\alpha}$). Because complex species are migrating faster than the polyelectrolyte, the equilibrium concentration of protein is related to that in the pure protein region by an expression analogous to Eq. (29). Upon replacement of migration rates by the corresponding retention times (proportional to their reciprocals), Eq. (29) becomes

30 $$\:{C}_{A}^{\alpha}\:=\:{C}_{A}^{b}\:\left[1\:+\frac{{t}_{A}({t}_{B}-{\overline{t}}_{B}}{{\overline{t}}_{B}({t}_{A}-{t}_{B}}\right]$$

which invalidates the consideration of $\:{C}_{A}^{b}$ as $\:{C}_{A}^{}$ unless $\:{\overline{t}}_{B}={t}_{B}$. Binding constants obtained thus far by frontal analysis capillary electrophoresis must therefore be classified as steady state rather than thermodynamic parameters ($\:{K}_{ss}$ cf. * K*).

### *Sedimentation velocity studies of heterogeneous associations*

In sedimentation velocity studies of rapidly reversible interactions between two macromolecular reactants the most likely situation to be encountered is that in which the 1:1 complex is the fastest migrating species ($\:{s}_{C}>{s}_{A}>{s}_{B}$). The predicted asymptotic concentration distribution (Gilbert and Jenkins 1959; Nichol and Ogston 1965a) comprises a reaction boundary (αβ) trailed by a β plateau of pure reactant, which can be either A or B depending on the ratio$\:\:{C}_{B}^{\alpha}/{C}_{A}^{\alpha}$. For an experimental system a variation in the initial mixing proportions may well result in a change from B to A as the slower-migrating boundary ‒ a situation that has been observed (Nichol and Winzor 1964) in a sedimentation velocity study of the electrostatic interaction between ovalbumin (A) and lysozyme (B) in phosphate buffer, pH 6.8, *I* 0.02 M.

The important point to note is that the concentration $\:{C}_{B}^{\beta}$ is again a steady-state parameter in that it does not equate with $\:{C}_{B}^{\alpha}$, its equilibrium concentration in the mixture. However, its combination with $\:{\overline{C}}_{A}^{}$, $\:{\overline{C}}_{B}^{}$, $\:{s}_{A}$, $\:{s}_{B}$ and $\:{\overline{s}}_{A}$ (the constituent sedimentation coefficient of the reaction boundary across which B does not disappear) allows the evaluation of $\:{C}_{A}^{\alpha}$ via the analog of Eq. (28), namely

31 $$\:{C}_{A}^{\alpha}=\left({\overline{C}}_{A}^{\alpha}-{\overline{C}}_{B}^{\alpha}+{C}_{B}^{\beta}\right)\:\frac{{\overline{s}}_{A}-{s}_{B}}{{s}_{A}-{s}_{B}}$$

**Fig. 8:**
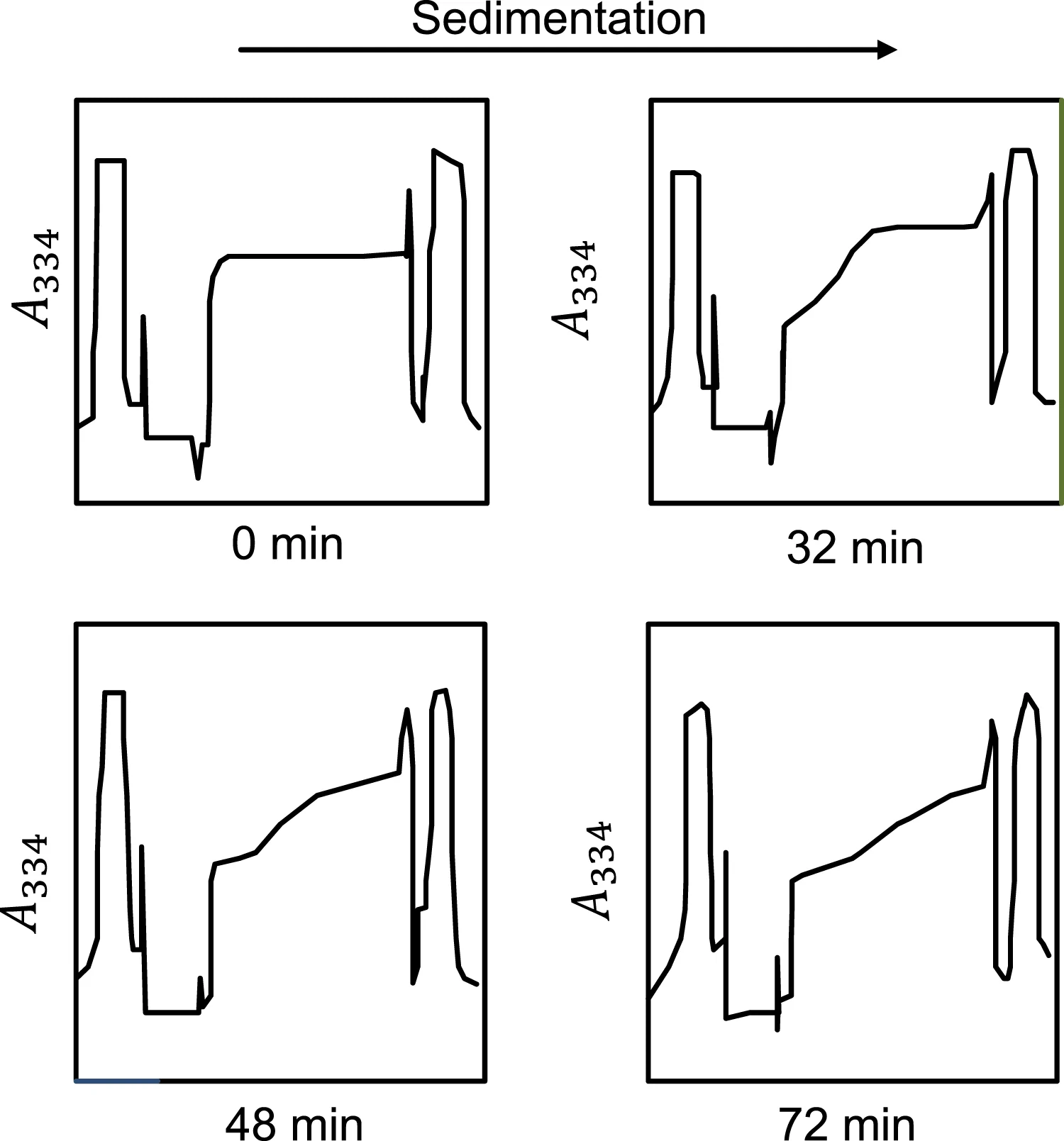
Sedimentation velocity patterns for a mixture of bovine serum albumin and methyl orange in 0.1 M phosphate buffer spun at 59,780 rpm for the indicated times. [Data taken from Fig. 6 of Steinberg and Schachman (1966).]

From Eq. (31) it is evident that the steady-state parameter $\:{C}_{B}^{\beta}$ equates with $\:{C}_{B}^{\alpha}$ only for systems with $\:{\overline{s}}_{A}={s}_{A}$, an identity reflecting the co-migration of faster-moving reactant and complex ($\:{s}_{C}={s}_{A}>{s}_{B})$. An example of that situation is illustrated in Fig. 8, which presents a spectrophotometric record (334 nm) of the progress of a sedimentation velocity experiment (59,780 rpm) on a mixture of bovine serum albumin (2 g/L) and methyl orange (0.49 mM) in 0.1 M phosphate buffer, pH 5.66 (Steinberg and Schachman 1966). For this interaction between a protein and a small ligand the concentration of methyl orange in the trailing boundary can be taken as its equilibrium concentration in the mixture provided that complex formation has no discernible effect on the sedimentation coefficient of the protein − a situation confirmed by the independence of $\:{\overline{s}}_{A}$ on mixing ratio [Tables II and III of Steinberg and Schachman 1966)]. This technique, termed sedimentation dialysis (Lloyd et al. 1968; Blake and Peacocke 1968), has been used to characterize the interactions of small ligands (proflavine and aminoacridines) with nucleic acids. However, identification of the concentration of the trailing ligand boundary ($\:{C}_{B}^{\beta})\:$as its equilibrium counterpart $\:{(C}_{B}^{\alpha})$ is undermined by the demonstration that complex formation between proflavine and deoxyribonucleic acid (Lloyd et al. 1968) results in a decreased sedimentation coefficient for the nucleic acid constituent. On the grounds that the system must therefore be categorized as one in which $\:{s}_{A}>{s}_{C}>{s}_{B}$, rather than $\:{s}_{A}={s}_{C}>{s}_{B}$, the concentration of the trailing boundary of pure ligand$\:{(C}_{B}^{\beta})$ was a steady-state parameter. Consequently, the evaluated binding constant ($\:{K}_{ss}$) was incorrectly identified as * K.*.

### Frontal size exclusion chromatography of heterogeneous association

Frontal size exclusion chromatography (Winzor and Scheraga 1963) not only affords the equivalent of ascending (advancing) and descending (trailing) migration patterns but also a reliable means of achieving ligand-binding situations wherein acceptor and acceptor−ligand complex(es) do comigrate by selecting a chromatographic matrix that excludes all forms of acceptor but allows partitioning of ligand. For small ligands this requirement has been met by choosing a tightly crosslinked gel such as Sephadex G-25 or BioGel P2 (Cooper and Wood 1968; Nichol et al. 1971, 1972; Ward and Winzor 1983); and the use of matrices with larger pore size has allowed the same procedure to be used with a protein (concanavalin A) as ligand and an even larger macromolecule (Dextran 2000) as acceptor (Hogg and Winzor 1985). However, the first ligand-binding study to be examined by the technique (Nichol and Winzor 1964) entailed frontal size-exclusion chromatography of ovalbumin−lysozyme mixtures on Sephadex G-100, a matrix into which both reactants partitioned. Recognition of the system as a situation involving the comigration of ovalbumin and its 1:1 complex with lysozyme therefore required the establishment of its conformity with the predicted theoretical migration behaviour of such a system (Gilbert and Jenkins 1959).

**Fig. 9:**
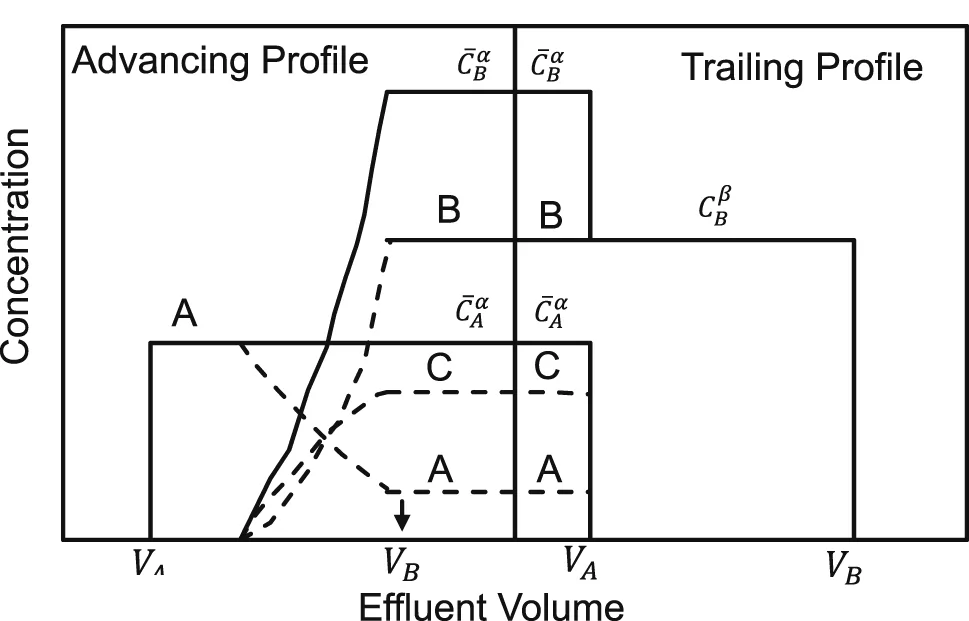
Theoretical asymptotic elution profiles for frontal chromatography of a reaction mixture when acceptor and acceptor−ligand complex comigrate ($\:{V}_{\mathrm{A}}={V}_{\mathrm{C}}>{V}_{B}$). Solid lines denote elution profiles in terms of total concentrations, and dashed lines the profiles for individual species (A, B and 1:1 complex C)

**Fig. 10:**
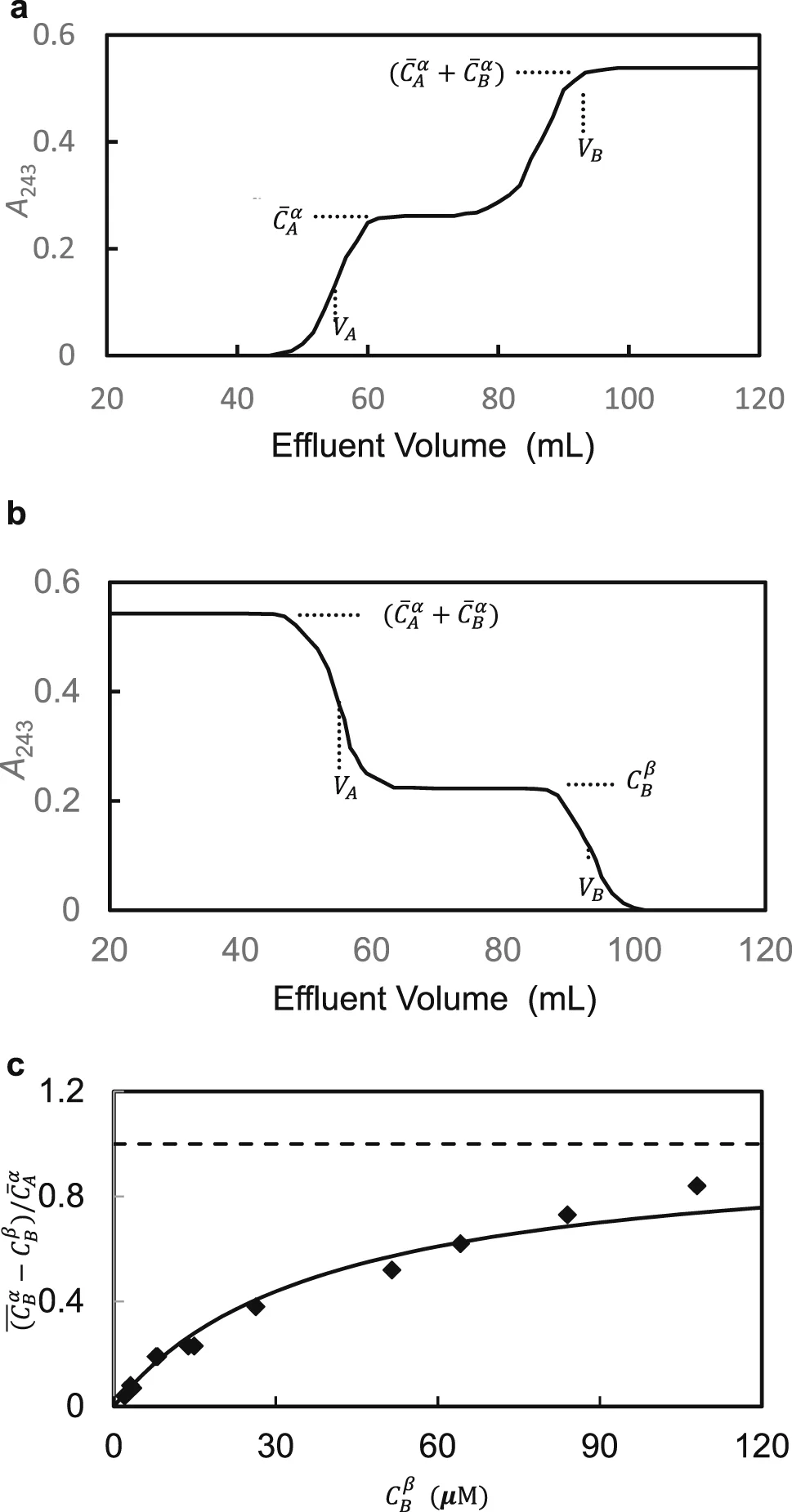
Frontal size-exclusion chromatography study of the 1:1 interaction between ovalbumin and lysozyme on Sephadex G-100 (pH 6.8, $\:{I}_{M}$ 0.02). (**a**) Advancing elution profiles for a mixture comprising 9.6 µM ovalbumin ($\:{\overline{C}}_{A}^{\beta}$) and 9.7 µM lysozyme ($\:{\overline{C}}_{B}^{\alpha}$) on Sephadex G-100 (pH 6.8, *I* 0.02 M). (**b**) The trailing elution profile. (**c**) Plot of the binding data from several such experiments in terms of a rectangular hyperbolic dependence upon $\:{C}_{B}^{\beta}={C}_{B}^{\alpha}$ [Data taken from Nichol and Winzor (1964).]

In size-exclusion chromatography elution volume is the counterpart of velocity in a single-phase system (Gilbert 1966; Nichol et al. 1967) − a change that has been incorporated into the predicted diffusion-free profiles (Gilbert and Jenkins 1959) for an interacting system with $\:{V}_{A}={V}_{C}>{V}_{B}$ (Fig. 9). In conformity with Eq. (31) and Fig. 8, the trailing (descending) elution profile comprises a faster-moving boundary at $\:{V}_{A}$ across which all acceptor disappears, followed by a slower-moving boundary of pure ligand with concentration $\:{C}_{B}^{}={C}_{B}^{}$ (the equilibrium concentration in the reaction mixture subjected to chromatography). Confirmation of the system as one exhibiting comigration of acceptor and acceptor−ligand complex comes from the form of the advancing (ascending) elution profile. Although the presence of a boundary of pure acceptor followed by an interaction boundary is also predicted for other velocity combinations (e.g., Fig. 6), the unique feature for the present system is the identity of the plateau concentration of free acceptor ($\:{C}_{A}^{b})$ with its total concentration in the mixture ($\:{\overline{C}}_{A}^{\alpha}$). Conformity of the advancing elution profile obtained (Nichol and Winzor 1964) in the experimental study of the ovalbumin−lysozyme interaction on Sephadex G-100 with this distinctive feature (Fig. 10a) justified the consideration of $\:{C}_{B}^{\beta}$ as $\:{C}_{B}^{\alpha}$ (Fig. 10b), and hence the thermodynamic characterization of the interaction in terms of a rectangular hyperbolic dependence of ($\:{\overline{C}}_{B}^{\alpha}$ ─ $\:{C}_{B}^{\beta\:}$) upon $\:{C}_{B}^{\beta\:}$ (Fig. 10c).

In view of the relative ease with which the co-migration of all acceptor species can be achieved by appropriate selection of the chromatographic matrix, frontal size-exclusion chromatography is the migration procedure of choice for characterizing acceptor−ligand interactions because the steady-state concentration of the pure-ligand plateau in the trailing elution profile $\:({C}_{B}^{}$) is also the equilibrium concentration.

### Steady-state dialysis

**Fig. 11:**
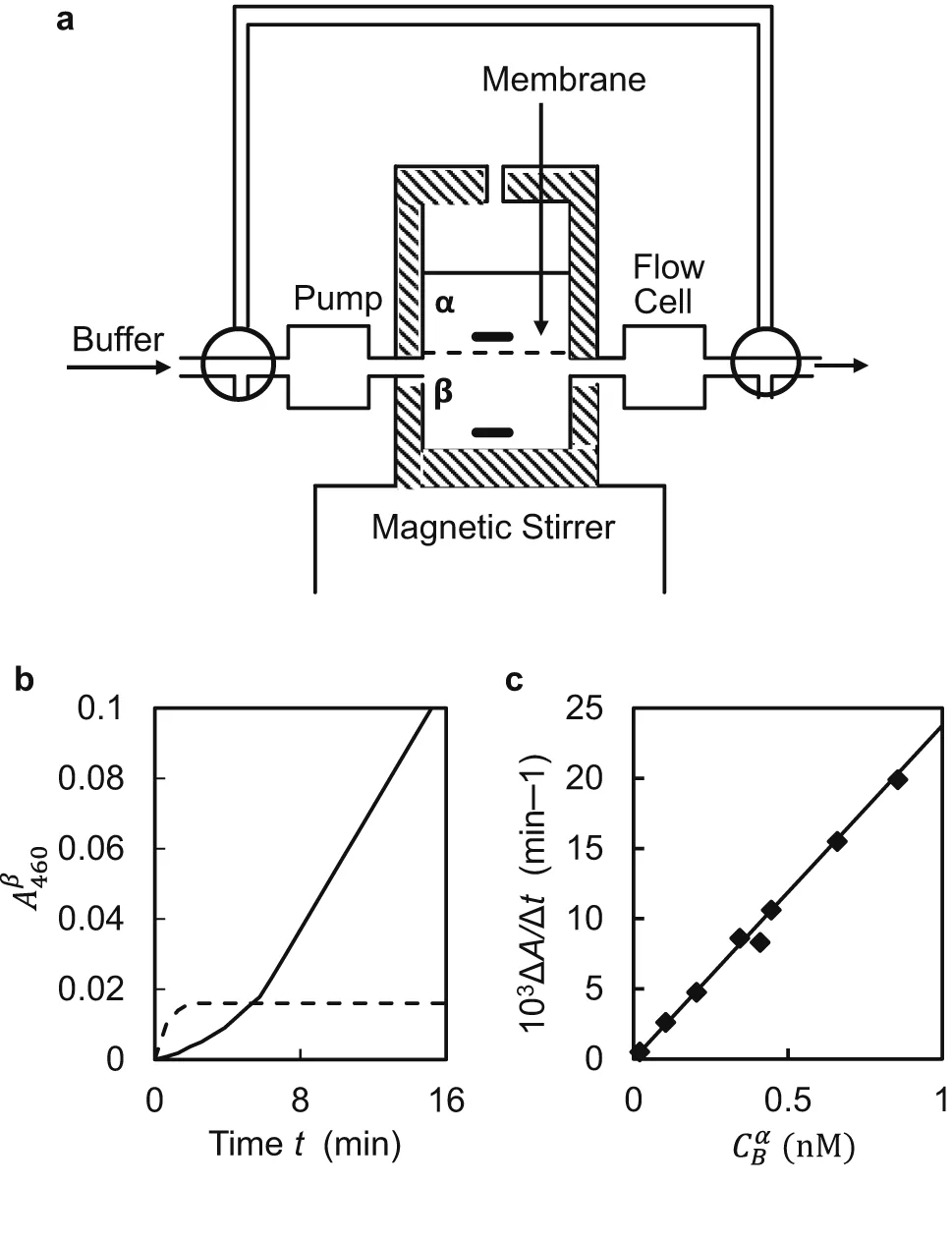
Studies of ligand binding by steady-state dialysis. (**a**) Schematic representation of the apparatus that incorporates the original technique (Colowick and Womack 1969) and a recycling variant thereof (Ford et al. 1983). (**b**) Time-course of the β-phase absorbance in calibration experiments with 0.52 mM methyl orange: original technique; recycling version. (**c**) Calibration plot for the evaluation of free ligand concentrations. [Data in (b) and (**c**) taken from Ford et al. (1983).]

We conclude this section by considering an experimental situation where a steady-state response was used to define its thermodynamic equilibrium counterpart ‒ a rate-of-dialysis technique designed (Colowick and Womack 1969) to overcome the lengthy time requirement inherent in the acquisition of ligand-binding data by equilibrium dialysis. The apparatus (Fig. 11a) comprised a dialysis cell with an upper compartment (α) containing the acceptor‒ligand mixture, separated by a semi-permeable membrane from a lower compartment (β) through which buffer (Colowick and Womack 1969) or recycled effluent (Ford et al. 1983) is pumped at a constant flow rate: the two taps in Fig. 11a are set for the original procedure. Provided that the liquid in both compartments is stirred to minimize concentration gradients in either phase, only a few minutes are required to establish a steady-state ligand concentration within the β compartment. As illustrated in Fig. 11b, the attainment of a steady state is manifested either as a time-independent concentration of ligand (Colowick and Womack 1969) or as a constant rate of concentration change in the recycling version of the technique (Ford et al. 1983). That steady-state response is then related to the free concentration of ligand in the upper compartment ($\:{C}_{B}^{\alpha}$) by calibrating the system with known concentrations of ligand (but no acceptor) in that compartment (Fig. 11c). To obtain binding data, acceptor‒ligand.

Mixtures with defined total reactant concentrations ($\:{\overline{C}}_{A}^{\alpha}$ $\:,{\overline{C}}_{B}^{\alpha})$ are placed in the upper compartment, and the corresponding free ligand concentration ($\:{C}_{B}^{\alpha})$ is inferred from the steady-state responses in the linear calibration plot. The magnitude of the binding constant for a 1:1 interaction then follows from Eq. (23) with $\:{C}_{C}$ taken as $\:({\overline{C}}_{B}-{C}_{B}$) and $\:{C}_{A}$ as $\:{\overline{C}}_{A}-{C}_{C})$. As an empirical means of determining free ligand concentrations in an acceptor‒ligand mixture the recycling variant (Ford et al. 1983) has no obvious advantages over the original rate-of-dialysis procedure (Colowick and Womack 1969). However, it does afford a means of obtaining better insight into the nature of the empiricism.

The recycling rate-of-dialysis assembly bears a striking resemblance to a later version (Stokes 1950) of the diaphragm cell devised by Northrop and Anson (1929) for the measurement of diffusion coefficients. For an experiment with initial solute concentration $\:({C}_{B}^{}{)}^{o}$ in the upper compartment, the concentrations of solute in the two phases at time *t* are related to its translational diffusion coefficient, $\:{D}_{B}$, by the expression (Northrop and Anson 1929; Gordon 1945)

32 $$D_{B} = \frac{1}{{\xi t}}\mathrm{ln}\frac{{(C_{B}^{\alpha } )^{o} }}{{C_{B}^{\alpha } - C_{B}^{\beta } }};\xi = \frac{{(A/\delta x)(V^{\alpha } + V^{\beta } )}}{{V^{\alpha } V^{\beta } }}$$

where *ξ* is a cell constant that depends not only on the thickness ($\:\delta{x})$ and effective pore area (* A*) of the membrane, but also the volumes ($\:{V}^{\alpha},{\:V}^{\beta}$) of liquid in the two phases (Renken 1955). On the basis that $\:{C}_{B}^{\alpha}=({C}_{B}^{\alpha}{)}^{o}-{C}_{B}^{\beta}{(V}^{}/{V}^{\alpha})$, Eq. (32) may be rewritten as

33 $$\:\frac{{C}_{B}^{\alpha}-{C}_{B}^{\alpha}[1+({V}^{\beta}/{V}^{\alpha}\left)\right]}{({C}_{B}^{\alpha}{)}^{o}}\:=\:\mathrm{exp}\left(\xi{D}_{B}t\right)\approx\:\:\:1-\xi{D}_{B}t$$

During the early stages of a recycling experiment the transport of solute across the membrane into the β-phase is sufficiently small for $\:{C}_{B}^{}$ to be essentially unchanged from its initial value, $\:({C}_{B}^{\alpha}{)}^{o}$. Under those circumstances the expression for solute concentration in the β-phase becomes

34 $$\:{C}_{B}^{\beta}\:=\:\left[\frac{({C}_{B}^{\alpha}{]}^{o}}{1+({V}^{\beta}/{V}^{\alpha})}\right]\xi{D}_{B}t$$

which, subject to constancy of the solute diffusion coefficient $\:{D}_{B}$, establishes the existence of a transient steady state with the slope of a linear time dependence, $\:{D}_{B}$, that is proportional to $\:({C}_{B}^{}{]}^{o}$. Sample calculations (Ford et al. 1983) have indicated that this situation should prevail for about 20 min in the recycling version of the rate-of-dialysis technique ‒ a prediction consistent with the experimental finding (Fig. 11c).

There have been many misgivings about the validity of the rate-of-dialysis procedure because of the empiricism associated with its development (Colowick and Womack 1969). However, the above theoretical considerations have justified use of the transient steady-state concentration $\:{C}_{B}^{\beta}$ to define the ligand concentration $\:{C}_{B}^{\alpha}$ in a mixture with composition [$\:({\overline{C}}_{A}^{\alpha})^{o}$ $\:({\overline{C}}_{B}^{\alpha}{)}^{o}$]. Concern about the validity of results obtained by this empirical rate-of-dialysis procedure is thereby allayed. This system differs from its above predecessors in that the attainment of a steady state was never in question: identification of the source of that steady state was the matter to be established.

## Zonal migration studies of ligand binding

**Fig. 12:**
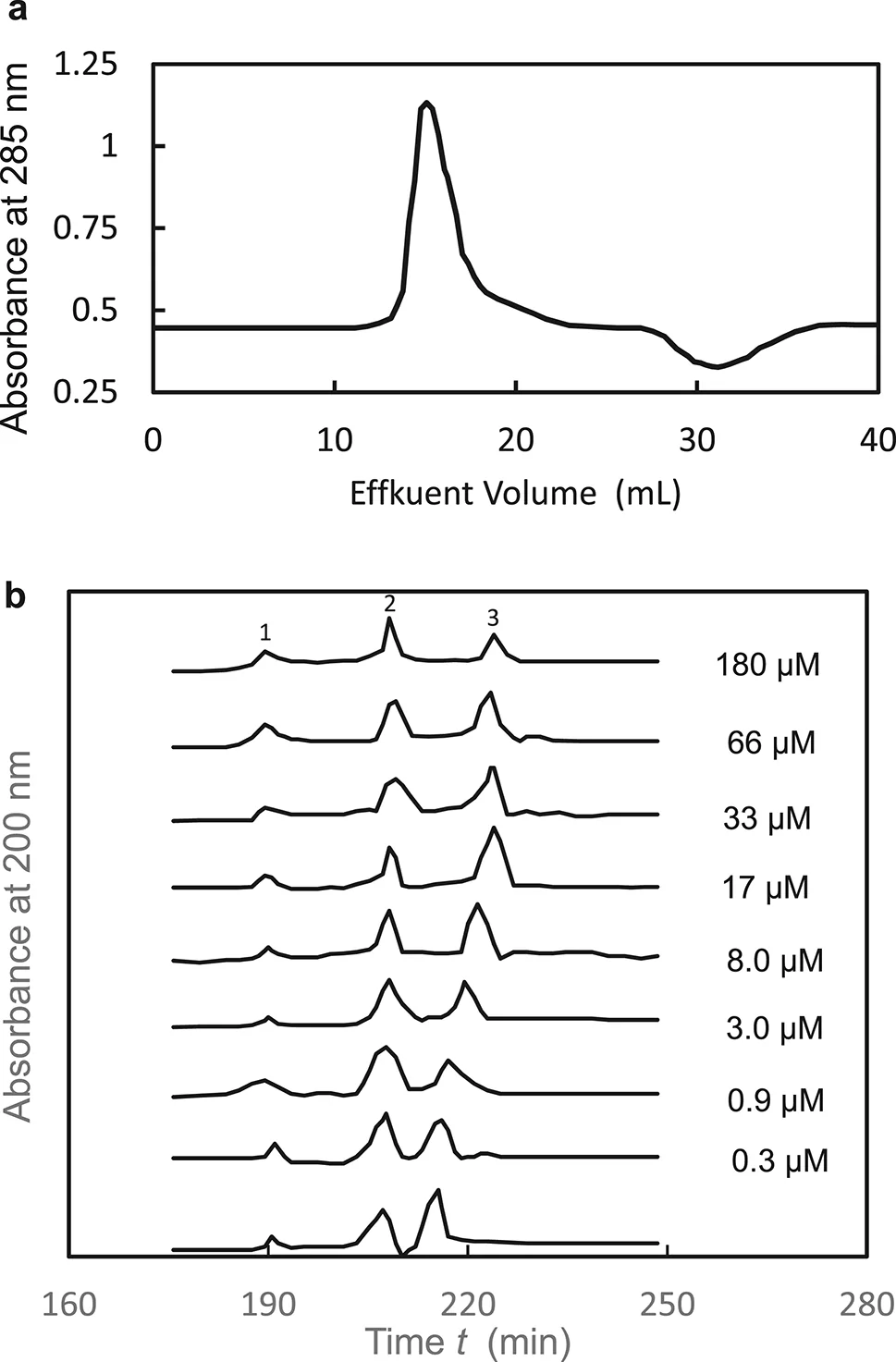
Examples of zonal studies of ligand interactions involving the passage of a small zone of acceptor through a medium preequilibrated with a known concentration of ligand. (**a**) Size-exclusion chromatography of ribonuclease on a Sephadex G-25 column preequilibrated with 2’-cytidylic acid. (**b**) Affinity capillary electrophoresis of carbonic anhydrase (peak 3) in the presence of buffer (pH 8.4) containing the indicated concentrations of 4alkylbenzenesulphonamide: no migration is observed for mesityl oxide (peak 1), a monitor of osmotic flow, or myoglobin (peak 2). [Data in (**a**) and (**b**) taken from Hummel and Dreyer (1962) and Chu et al. (1992) respectively.]

Zonal techniques are at a decided disadvantage in the study of protein−ligand interactions in rapid association equilibrium because of the dissociation that inevitably occurs during the separation of reactant and complex species. This problem can be averted to some extent by subjecting a small zone of acceptor solution to chromatography or electrophoresis in a medium pre-equilibrated with a known concentration of ligand. A faster migration rate of protein species results in continual movement of the applied zone into a region of the column with a known concentration of ligand, with which the protein re-establishes equilibrium (or a steady state). This approach was developed in the context of size-exclusion chromatography by Hummel and Dreyer (1962), who analysed elution profiles for a zone of ribonuclease on a column of Sephadex G-25 pre-equilibrated with a range of concentrations of 2’-cytidylic acid. In the resulting elution profile (Fig. 12a) the increased absorbance of the eluted protein zone reflects the combination of contributions from protein and bound ligand, whereas the subsequent trough (at the elution volume of ligand $\:{V}_{B}$) quantifies the amount of ligand taken up by the protein zone as the result of reversible complex formation.

The same principle is employed in affinity capillary electrophoresis studies of protein interactions with charged ligands (Chu et al. 1992; Chu and Whitesides 1992; Honda et al. 1992; Kuhn et al. 1994), where the extent of complex formation with the pre-equilibration concentration of ligand is monitored by the effect of $\:({C}_{B}{)}_{p}$ on the protein migration rate (quantified as a retention time for the zone to migrate a fixed distance). An example of this approach (Chu et al. 1992) is presented in Fig. 12b, which shows the dependence of the retention time for bovine carbonic anhydrase (peak 3) on the concentration (µM) of 4-alkylbenzenesulphonamide included in the Tris−chloride buffer (pH 8.4). No variation in retention time is observed for mesityl oxide (peak 1), which is a monitor of electroosmotic flow, or for human heart myoglobin (peak 2), a noninteracting protein.

In view of the demonstrated need to distinguish between the attainment of a kinetic steady state rather than thermodynamic equilibrium in frontal migration studies, the same precautionary approach clearly needs to be adopted for these zonal counterparts.

### The Hummel−Dreyer approach

The zonal gel chromatographic procedure developed by Hummel and Dreyer (1962) to characterize ligand binding quickly became popular as a rapid method for determining the stoichiometry and strength of interactions between small ligands and macromolecular acceptors such as proteins. In that regard the above discussions of frontal procedures establish that the procedure entails an implicit assumption that the faster-migrating reactant (A) and complex(es) C co-migrate. Although this requirement is met in situations where the acceptor is excluded from the gel phase (Hummel and Dreyer 1962; Fairclough and Fruton 1966), it may well cease to apply in systems where the acceptor also partitions into the gel phase. Such a situation has been encountered in an application of the Hummel−Dreyer protocol to characterize metal−nucleotide interactions by size-exclusion chromatography on Sephadex G-10 (Colman 1972).

As the small zone of acceptor A transverses the column, it removes ligand B from the gel phase via complex formation until a steady state is attained in which the composition of the migrating zone is determined by the laws of mass conservation and mass action operating in concert. Mass conservation considerations dictate that the area of the trough in Fig. 12a quantifies the amount of complexed ligand in the acceptor zone. However, its magnitude also accommodates any departures in free ligand concentration from the pre-equilibration concentration $\:{(C}_{B}{)}_{p}$ within the acceptor zone effected by differences between the migration rates (elution volumes) of A and C.

**Fig. 13:**
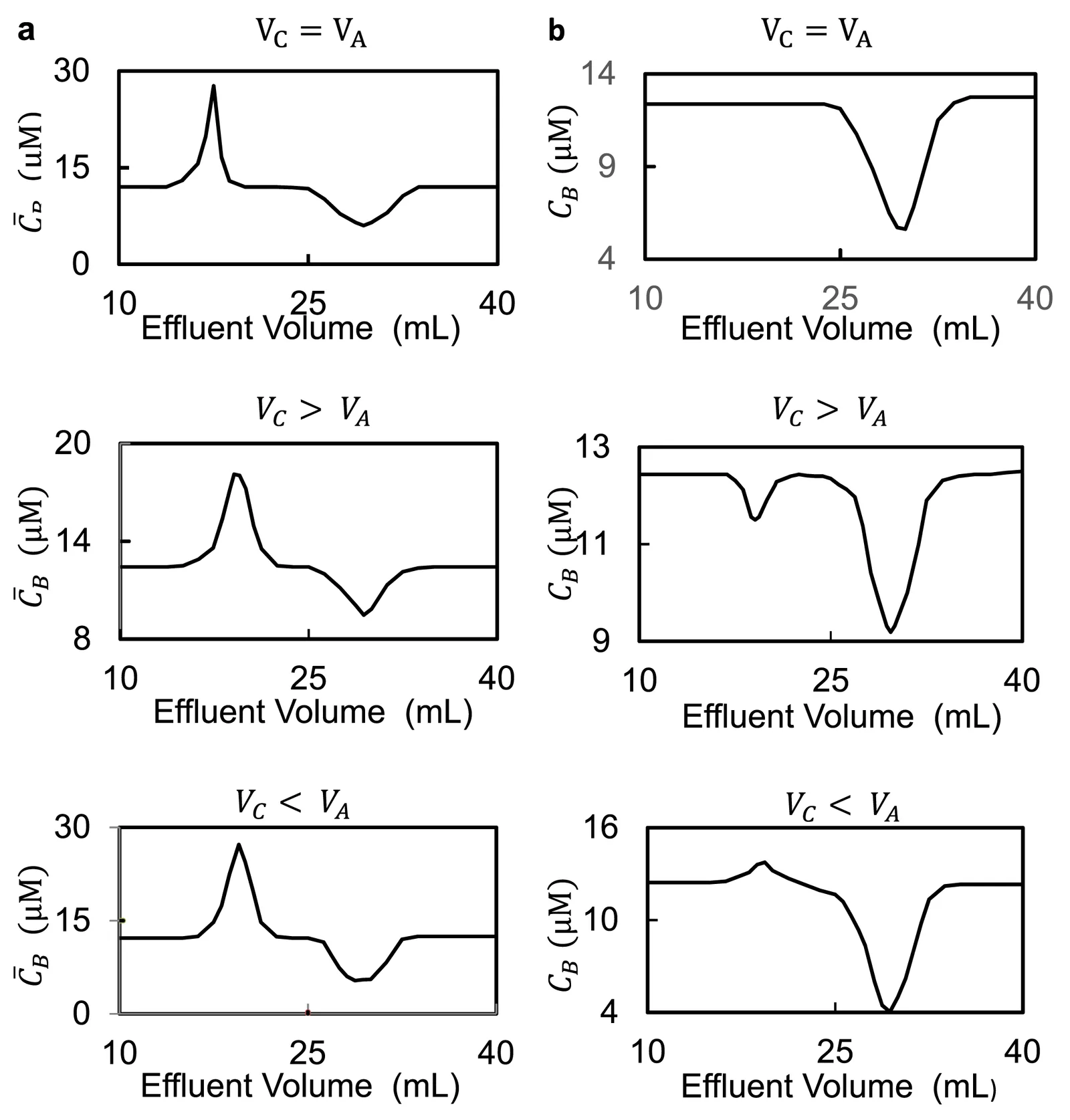
Simulated Hummel‒Dreyer elution profiles for ligand interaction with acceptor, A + B ⇄ C, governed by an association constant of 80,000 M-1. (**a**) Elution profiles for total ligand concentration $\:{\overline{C}}_{B}$. (**b**) Elution profiles for free ligaand $\:{C}_{B}$. The uppermost panels reflect the original situation (Hummel and Dreyer 1962) in which acceptor and complex comigrate ($\:{V}_{C}={V}_{A})$, whereas the centre and lowest panels refer to systems with $\:{V}_{C}>{V}_{A}$ and $\:{V}_{C}<{V}_{A}$ respectively. [Data taken from Fig. 1 of Cann et al. (1989).]

The existence of departures in the free ligand concentration from its pre-equilibration value, $\:{(C}_{B}{)}_{p}$, has been established (Cann et al. 1989) by numerical simulation of Hummel−Dreyer elution profiles for the total and free concentrations of ligand (Fig. 13a and b respectively). The uppermost patterns, which describe the situation where A and C co-migrate, confirm the identity of $\:{C}_{B}$ and $\:{(C}_{B}{)}_{p}$; and hence the validity of calculating the amount of complexed ligand from the area of the trough at $\:{V}_{B}$. Alternatively, in instances conducive to the delineation of elution profiles for the constituent concentrations of both reactants ($\:{\overline{C}}_{A}$ and $\:{\overline{C}}_{B}$) multiple estimates of a steady-state binding function $\:{\nu}_{ss}$ (mol ligand bound/mol acceptor),

35 $$\:{\nu}_{ss}=\:\frac{{{\overline{C}}_{B}}-{(C}_{B}{)}_{p}}{{\overline{C}}_{A}}$$

can be obtained at elution volumes within the acceptor zone (Colman 1972). For a system with $\:{V}_{A}={V}_{C}$, this steady state binding function is also the thermodynamic parameter because $\:{(C}_{B}{)}_{p}$ is also $\:{C}_{B}$.

Numerical simulations of Hummel−Dreyer elution profiles for $\:{\stackrel{-}{C}}_{B}$ and $\:{C}_{B}$ in the situation where the complex migrates more slowly than the acceptor ($\:{V}_{C}$ > $\:{V}_{A}$) are shown in the centre frames of Fig. 13, where the existence of a trough in free ligand concentration across the acceptor zone clearly invalidates the consideration of $\:{\nu}_{ss}$ as its thermodynamic counterpart. In studies of the interaction between ATP and magnesium ions (Colman 1972), to which this combination of elution volumes applies (Cann et al. 1989), the steady-state binding function defined by Eq. (35) clearly underestimates the extent of ligand binding. Indeed,

36 $$\:{\nu}_{ss}=\nu+\:\frac{{{\overline{C}}_{B}}-{(C}_{B}{)}_{p}}{{\overline{C}}_{A}}$$

where $\:{\overline{C}}_{A}$ denotes the total acceptor concentration at the effluent volume to which $\:{C}_{B}$ refers. A steady-state association constant $\:{K}_{ss}$ for a 1:1 interaction may thus be determined from the expression

37 $$\:{K}_{ss}=\:\frac{{V}_{ss}}{\left(1-{V}_{ss\:}\right)({C}_{B}{)}_{p}}$$

which exhibits a dependence on total acceptor concentration $\:{\overline{C}}_{A}$ and attains a limiting value, $\:{K}_{ss}^{}$, as $\:{\overline{C}}_{A}$   →0. That extrapolated value is related to the thermodynamic association constant * K* by (Cann et al. 1989)

38 $$\:{K}_{ss}^{\infty}\:=\:K\:\frac{({V}_{B}-{V}_{C})}{({V}_{B}-{V}_{A})}\:$$

On this basis the extrapolated steady-state constant for the ATP−Mg^2+^ system (Colman 1972) would have underestimated * K* by 22% based on the measured values of 27.5 mL for $\:{V}_{B},\:16.3$ mL for $\:{V}_{C}$ and 18.8 mL for $\:{V}_{A}$ reported in Table 1 of Cann et al. 1989) for the same system.

**Fig. 14:**
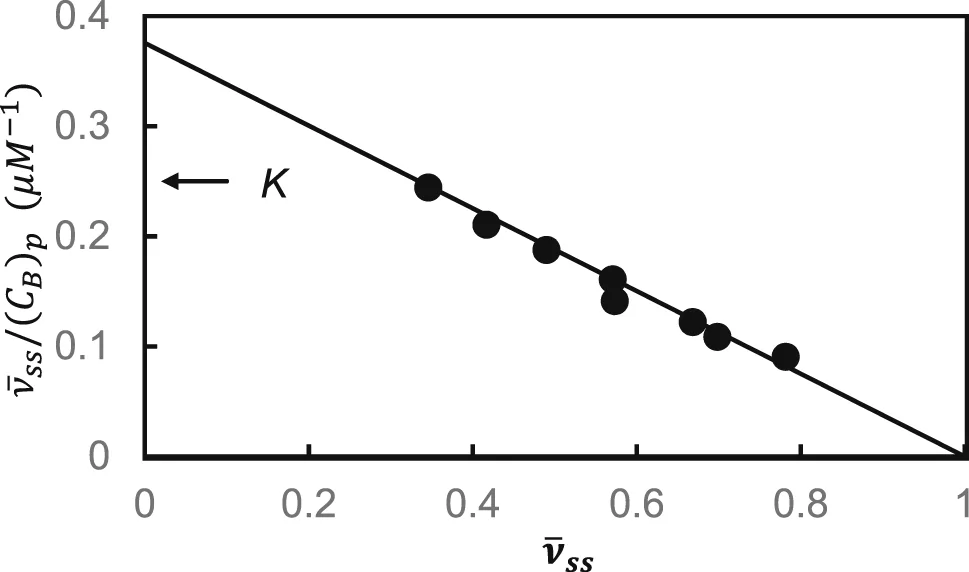
Scatchard plot of steady-state binding data [$\:{(C}_{B}{)}_{p},\:\overline{\nu}_{ss}$] obtained by the application of Eq. (35) to results from a Hummel‒Dreyer study on Sephadex G-75 of the interaction between soybean trypsin inhibitor and cytochrome *c* (pH 6.8, *I* 0.01 M). [Data taken from Fig. 5 of Cann et al. (1989).]

From Eq. (38) it is evident that $\:{K}_{ss}$ should overestimate * K* in situations where the complex migrates faster than acceptor ($\:{V}_{C}$ < $\:{V}_{A})$ − a prediction verified by numerical simulations (lowest panels in Fig. 13), which show that $\:({C}_{B}{)}_{p}$ underestimates $\:{C}_{B}$ across the acceptor zone. Confirmation of that prediction has also been provided (Cann et al. 1989) by employing the zonal protocol to obtain data for the binding of cytochrome *c* to soybean trypsin inhibitor by size-exclusion chromatography on Sephadex G-75 under conditions identical with those used for its experimental characterization by frontal chromatography (Cann and Winzor 1987). Use of Eq. (35) to calculate the steady-state binding function from measurements of $\:{\overline{C}}_{B}$ and $\:{\overline{C}}_{A}$ across acceptor zones in experiments with $\:({C}_{B}{)}_{\mathrm{p}}$ in the range 1.4 to 8.5 µM yielded the Scatchard plot shown in Fig. 14: $\:{\overline{\nu}}_{ss}$, the average of values for a given $\:{(C}_{B}{)}_{\mathrm{p}}$, corresponds to the parameter deduced from the area of the trough in Fig. 13b;. Extrapolation of the linear plot to the abscissa and ordinate axes establishes 1:1 stoichiometry for the interaction and a steady-state association constant (essentially $\:{K}_{ss}^{\infty}$) of 3.7 (± 0.6) x 10^5^ M^−1^, which is larger than the thermodynamic association constant * K* of 2.5 (± 0.7) ⋅ 10^5^ M^−1^ obtained from the frontal chromatography study (Cann and Winzor 1987). However, as predicted by Eq. (40), its combination with the three species elution volumes ($\:{V}_{A}$ = 35.5, $\:{V}_{B}$ = 45.7, $\:{V}_{C}$ = 32 mL) leads to an estimate of 2.8 (± 0.5) ⋅ 10^5^ M^−1^ for * K*, which essentially duplicates the earlier thermodynamic value.

The above considerations also apply to counter ion electrophoresis (Ueng and Bonner 1979), a gel electrophoresis technique which also yields a Hummel−Dreyer distribution in total ligand concentration, but with electrophoretic mobility as the abscissa (Ueng and Bonner 1979; Cann and Fink 1985). After the anode chamber has been filled with labelled Ca^2+^−supplemented buffer, a small zone of cationic Ca^2+^−binding protein is placed at the cathode end of the polyacrylamide gel before the application of electric field.* E*. A uniform concentration $\:{(C}_{B}{)}_{p}$ of Ca^2+^ ion is quickly created within the gel column, through which cationic protein migrates to generate a pattern analogous to the profiles in Fig. 13a with electrophoretic mobility substituted for * V* (Ueng and Bonner 1979, Cann and Fink 1985). On the grounds that the binding of calcium to the cationic protein yields a complex with lower mobility, the middle patterns in Fig. 13 are the appropriate reference. Indeed, because the electrical filed is applied for a fixed time before sectioning of the gel to quantify the total ligand (and acceptor) concentration distributions, the abscissa may be defined in terms of * x*, the distance migrated in that fixed time *t*. From Fig. 13b it is evident that the evaluation of a binding function as $\:({\overline{C}}_{B}-({C}_{B}{)}_{p}/{\overline{C}}_{A}$ gives rise to the steady-state parameter $\:{\nu}_{ss}$, an underestimate of its thermodynamic counterpart, *ν*, because of the trough in $\:{C}_{B}$ within the protein zone.

In summary, the Hummel−Dreyer approach for the characterization of ligand binding only yields an equilibrium distribution in situations where the acceptor and acceptor‒ligand complex(es) comigrate. In all other situations a steady-state ligand distribution is generated. Although this required combination of migration rates is often achievable in size-exclusion chromatography by the selection of a gel phase that excludes acceptor, it never arises in counter ion electrophoresis.

### Affinity capillary electrophoresis

From the foregoing discussion of Hummel−Dreyer systems the ligand distributions obtained in affinity capillary electrophoresis experiments (Fig. 12b) necessarily conform with the lowest patterns of Fig. 13a, b because of the faster migration of the acceptor−ligand complex(es): $\:{C}_{B\:}$is therefore greater than $\:{(C}_{B}{)}_{p}$ within the migrating protein zone. A binding function derived from ligand distributions would therefore be the steady-state parameter $\:{\nu}_{ss}$. However, in affinity capillary electrophoresis the experimental binding function is calculated from the extent of acceptor complex formation deduced from the weight-average mobility $\:\overline{u}$ of acceptor. For 1:1 complex formation the average electrophoretic mobility is given by

39 $$\bar{u} ~ = \frac{{u_{c} C_{C} + u_{A} C_{A} }}{\bar{C}} = \frac{{u_{C} C_{C} + u_{A} ({\bar{C}} - C_{C} )}}{\bar{C}}$$

from which the binding function ν may be determined as

40 $$\nu= \frac{{C_{C} }}{\bar{C}}= \frac{{\bar{u} - u_{A}}}{{u_{C} - u_{A} }}$$

The binding function obtained from mobility measurements is thus a thermodynamic parameter. However, the subsequent calculation of a binding constant as

41 $$\:{K}_{app}=\:\frac{\nu}{(1-\nu){(C}_{B}{)}_{p}}$$

disregards the disparity between $\:{C}_{B}$ and $\:{(C}_{B}{)}_{p}$ within the acceptor zone effected by the non-identity of $\:{u}_{A}$ and $\:{u}_{C}.$ For experiments with $\:{\overline{C}}_{A}$ $\:\ll\:{(C}_{B}{)}_{p}$ the magnitude of $\:{K}_{app}$ should be a reasonable estimate of the thermodynamic binding constant because of the relatively small difference between $\:{\overline{C}}_{B}$ and $\:{(C}_{B}{)}_{p}$ as the result of ligand uptake by the acceptor. Indeed, advantage had already been taken of this procedure much earlier in the characterization of noncovalent phosphate binding to ovalbumin by zonal polyacrylamide gel electrophoresis (Ward and Winzor 1981).

In affinity capillary electrophoresis migration is monitored in terms of retention times (Fig. 7b) which are proportional to the reciprocal of the corresponding mobility. With those substitutions the expression for the thermodynamic binding function, Eq. (40), becomes

42 $$\nu=\:\frac{(\overline{t}-{t}_{A})}{\left({t}_{C}-{t}_{A}\right)}\left(\frac{{t}_{C}}{\overline{t}}\right)$$

which is in accord with the approach used (Honda et al. 1992) in a study of lactobionic acid interaction with *Ricinus communis* agglutinin: the reported binding constant is the intrinsic association constant for interaction with two equivalent and independent sites on the dimeric agglutinin (Winzor 1995). Omission of the ($\:{t}_{C}/\overline{t}$) term from Eq. (42) in other studies (Chu et al. 1992; Chu and Whitesides 1992) leads to additional error in the evaluated binding function. However, such use of $\:(\overline{t}-{t}_{A})/({t}_{C}-{t}_{A})$ to define $\:\nu$ in Fig. 12b only leads to a 4% underestimate of the binding function for the lowest $\:{(C}_{B}{)}_{p}$, where the discrepancy would have been maximal.

As in their frontal counterparts, zonal studies of ligand binding yield patterns that are steady-state concentration distributions except for the special circumstance that the acceptor and acceptor−ligand complex(es) comigrate.

## Nonspecific binding of a protein to a linear polymer chain

Studies of ligand binding by mass migration methods has dominated discussions thus far. In a change of direction, we now illustrate the need to consider the possible establishment of a kinetic steady state even in static experiments where no mass migration occurs.

Consideration is first given to the situation in difference spectrophotometry, where the change in absorbance at a given wavelength is traditionally interpreted in terms of rapidly attained thermodynamic equilibrium. Although such treatment of ligand binding data obtained by difference spectroscopy is certainly valid for small univalent ligands, there is potential for a transitory kinetic steady state to be established as a precursor to equilibrium attainment when the ligand is macromolecular. This possibility is illustrated in general terms before its illustration and interpretation with studies of the thrombin−heparin interaction.

The binding of proteins to polymer chains comprising identical or very similar units often entails interaction of the protein (ligand, B) with a linear sequence of units on the polymer (acceptor, A) chain. Examples include the binding of proteins to polynucleotides (Latt and Sober 1967; McGhee and von Hippel 1974), to polysaccharides (Olson et al. 1991; Munro et al. 1998, 2000), and also to the actin‒tropomyosin‒troponin complex (Greene and Eisenberg 1980). Thermodynamic characterization of such systems is complicated because the similarity of repeat units of the polymer chain creates a situation whereby any residue sequence of appropriate length is a potential binding site. Absence of specific acceptor sites has led to the description of the protein−polymer interaction as nonspecific binding, even in instances where a single intrinsic association constant (Klotz 1946) suffices to describe the system. Because the random attachment of protein to linear segments of the polymer chain can lead to suboptimal alignment of ligand molecules on the acceptor chain, additional dissociation and reassociation events are required to achieve the ultimate arrangement that reflects the attainment of thermodynamic equilibrium. This impediment to rapid equilibrium attainment has been designated as the parking problem (Hill 1978; Hill et al. 1980).

For equivalent and independent binging of ligand (B) to a sequence of *n* units of a linear sequence comprising *N* such units, the general form of the binding equation for *ν*, the molar ratio of bound ligand to total acceptor is (Klotz 1946)

43 $$\nu=\:\frac{\sum\:_{1}^{{Q}\:}{i}{{C}}_{{i}}^{{h}}{{K}}^{{i}}{{C}}_{{B}}^{{i}}}{1+\sum\:_{1}^{{Q}}{{C}}_{{i}}^{{h}}{{K}}^{{i}}{{C}}_{{B}}^{{i}}}$$

where $\:{C}_{B}$ is the free lignd concentration, Q the integer value of *N*/n, and where $\:{\mathrm{C}}_{i}^{h}=h!/[i!\left(h-i\right)!]\:$is the number of possible arrangements of the *h* items (bound ligand and unoccupied units) available for distribution in the lattice. In the attachment of the *i*th ligand molecule to an *N*-unit acceptor lattice the number of items to be arranged is * h=N-ni+i*, whereupon (Latt and Sober 1967)

44 $$\:{\mathrm{C}}_{i}^{h}=\:\frac{\left(N-ni+i\right)!}{i!\left(N-ni\right)!}$$

That﻿ combinatorial approach can be extended (Kelly et al. 1976; Epstein 1979) to encompass the possibility that a large ligand such as a protein may bind to *n* units but cover a larger number (*m*) by substituting $\:[m\left(i-1\right)+n]$ for *ni* in Eq. (44). The number of possible arrangements of the *h* items available for distribution in the lattice then becomes

45 $$\:{\mathrm{C}}_{i}^{h}=\:\frac{\left[N-m\left(i-1\right)-n+i\right]!}{\left[N-m\left(i-1\right)+n\right]!i!}$$

**Fig. 15:**
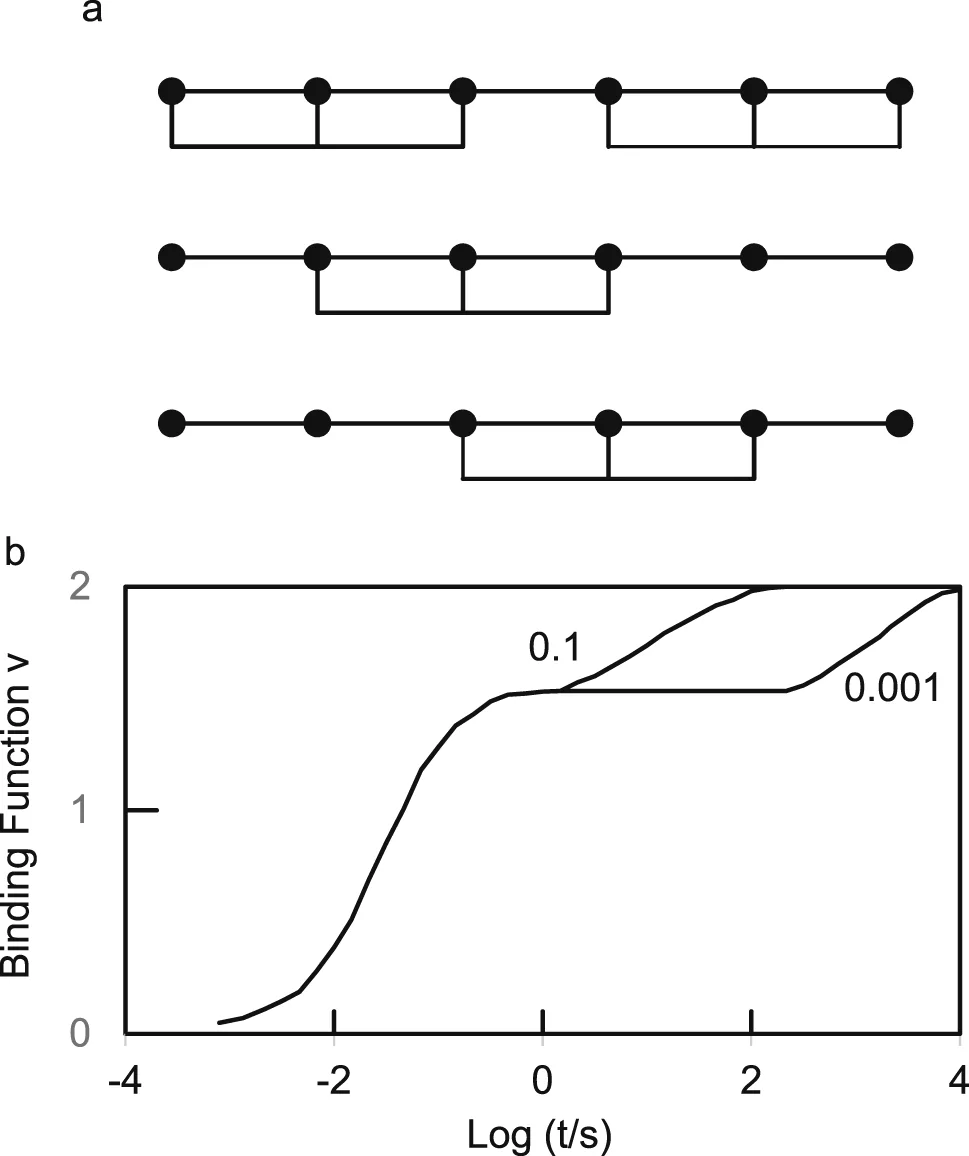
Potential for steady-state attainment in the nonspecific binding of a protein to a linear lattice of identical sites. (**a**) Examples of initial lattice saturation during random attachment of a protein to a sequence of three sites on a six-residue linear lattice: the uppermost example reflects attainment of the thermodynamic endpoint (*ν* = 2), whereas the other two are transient kinetic states (with ν = 1) because of suboptimal attachment of the first molecule. (**b**) Logarithmic time-dependence of the approach to the thermodynamic endpoint for the same system based on kinetic simulations with $\:{\overline{C}}_{A}=1\:\mu{M},\:{\overline{C}}_{B}=100\:\mu{M},\:{k}_{f}=0.1\:{\mu{M}}^{-1}{\mathrm{s}}^{-1}$ and the indicated values ($\:{s}^{-1}$) for the dissociation rate constant $\:{k}_{r}$. [Data in (**b**) taken from Munro et al. (1998).]

The parking problem is illustrated in Fig. 15a by considering the binding of a large ligand to three-residue sequences on a six-unit linear lattice. Although there are two possible ways of initial ligand binding conducive to the attachment of a second ligand molecule (a $\:{\nu\:}_{max}$of 2), there are also two situations in which random ligand attachment leads to seeming acceptor saturation with only one ligand molecule attached. A transient (steady state) situation therefore exists with$\:{\nu\:}_{ss}$ = 1.5 until dissociation and reassociation events allow the optimal (thermodynamic equilibrium) arrangement with $\:{\nu}_{max}$= 2 to be achieved. Because the rate of dissociation is the dominant factor in the repositioning of ligand molecules on the acceptor lattice, the lifetime of that steady state can be considerable (Munro et al. 1998).

Relative stability of the steady state is evident from Fig. 15b, which summarizes the results of kinetic simulations with respective total acceptor and ligand concentrations of 1 µM and 100 µM for the above system (Fig. 15a) in which the binding of ligand to three-residue sequences on the six-unit acceptor lattice is governed by an association rate constant of 10^5^M^−1^s^−1^and dissociation rate constant ranging between 0.1 and 0.001 s^−1^. These systems with intrinsic binding constants of 10^6^ to 10^8^M^−1^ attain the steady-state ($\:{\nu\:}_{ss}$= 1.5) within a second, and thermodynamic equilibrium is attained within 2 min for the weakest-affinity system. Although the parking problem should therefore be of little concern in identifying the equilibrium stoichiometry for this system, considerably more patience would be required on the part of a researcher to avoid misidentification of the steady-state response as the equilibrium value for the highest-affinity system, which is only attained after more than 24 h (Munro et al. 1998).

### The interaction of thrombin with heparin

Heparin is a sulphated glycosaminoglycan in which the repeat unit is a disaccharide comprising α-L-iduronic acid-2-sulphate linked β-1,4 to N-sulphurylglucosamine-6-sulphate, which exerts its therapeutic effect by catalysing the antithrombin-mediated inhibition of various coagulation proteases (Bjőrk and Lindahl 1982). Initial enthusiasm for quantitative characterization of the interaction between thrombin and heparin (Evington et al. 1986; Nesheim et al. 1986) waned with the realization (Olson et al. 1991) that heparin does not possess specific sites for thrombin, which merely binds electrostatically to the highly charged polyanion.

**Fig. 16:**
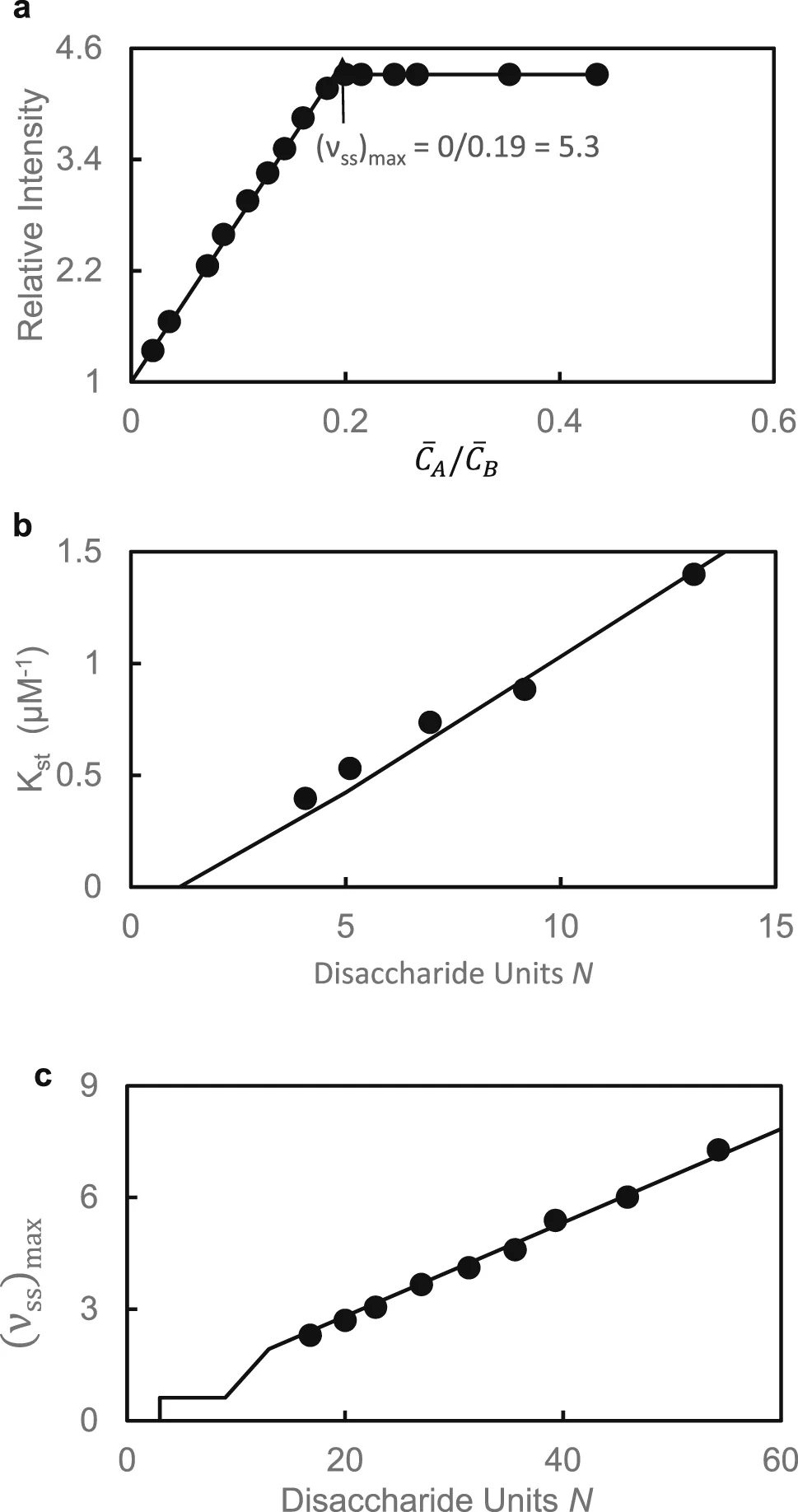
Stoichiometry of the nonspecific interaction between thrombin and disaccharide units on heparin. (**a**) Spectrophotometric titration of dansylated thrombin with a heparin sample comprising 45 disaccharide units. (**b**) Determination of the size of the binding segment on heparin by quantitative affinity chromatography: the abscissa intercept defines the magnitude of (*n* ‒ 1). (**c**) Dependence of the heparin capacity upon chainlength *(∙)* as well as its predicted counterpart for enzyme binding to two but covering six disaccharide units (^**______**^). [Data in (**a**) and (**c**) taken from Nesheim et al. (1986); those in (**b**) from Olson et al. (1991).]

The most extensive investigation of reaction stoichiometry for the thrombin−heparin interaction was a spectrofluorometric study (Nesheim et al. 1986) involving the titration of a fixed amount of dansylated thrombin (1.5 mL, 0.68 µM) with heparin That spectrofluorimetric titration for a 27,400 kDa heparin sample is shown in Fig. 16a, where the total heparin concentration $\:({\overline{C}}_{A}$) is expressed in terms of disaccharide content ($\:N={M}_{A}/615)$. The titration endpoint was originally interpreted in terms of the number of disaccharide units comprising a specific thrombin-binding site (Nesheim et al. 1986) but becomes the estimate of its steady-state counterpart, $\:({\nu}_{ss}{)}_{max}$, when the binding is nonspecific. From Fig. 16a, it seems likely that the value of 5.3 for $\:({\nu}_{ss}{)}_{max}$ would then imply thrombin occupancy (coverage) of a sequence of six disaccharide units * (m=6)*. The existence of a smaller value for *n* (the number of units to which thrombin actually binds) comes from measurements (Olson et al. 1991) of the binding constant by quantitative affinity chromatography of thrombin on immobilized heparin in experiments where the combination of high heparin and low thrombin concentrations ensures a very low extent of acceptor saturation (ν < 1) and hence equilibrium attainment (Olson et al. 1991). Because there are * (N-n+1)* ways of making the first ligand attachment [numerator of Eq. (44) with *i* = 1], the measured (stoichiometric) binding constant for 1:1 interaction $\:{K}_{ST}$ is related to its intrinsic counterpart *K* (Klotz 1946) by the relationship

46 $$\:{K}_{ST}=\left(N-n+1\right)K$$

A value of * n* can thus be inferred from the abscissa intercept (* n-1*) of the dependence of $\:{K}_{st}$ upon heparin chainlength * N*. The value of unity for that abscissa intercept of data (Olson et al. 1991) shown in Fig. 16b signifies a disaccharide sequence as the heparin binding site for chemical interaction with. thrombin. An intrinsic association constant of 1.2 x 10^5^ M^−1^ s^−1^ emanates from the slope, (* N-1)K*.

Measurements of the dependence of heparin capacity upon polysaccharide chainlength (Nesheim et al. 1986) are summarized *(∙)* in Fig. 16c, together with its theoretical counterpart (^_____^) for the situation involving the binding of thrombin to two (* n=2)* but coverage of six disaccharide units * (m=6)* − a model in keeping not only with the conclusion that chemical interaction between thrombin and heparin is restricted to two disaccharide units (Gan et al. 1994) but also with the crystal structure for thrombin bound to a heparin octasaccharide (Carter et al. 2005).

Results obtained at low levels of acceptor saturation do reflect equilibrium binding, but their quantitative interpretation requires knowledge of the stoichiometry of the interaction between a protein with a linear lattice of acceptor sites. That information needs to be obtained from mixtures with high levels of lattice saturation, conditions commensurate with existence of the parking problem and its inherent introduction of a kinetic steady state en route to thermodynamic equilibrium.

### Static light scattering of nonassociating solutes

To conclude these deliberations on the need for distinction between the attainment of thermodynamic equilibrium or merely a kinetic steady state, we consider the situation pertaining in static light scattering measurements on nonassociating macromolecular solutes. In this instance problems arose in interpreting the concentration dependence of turbidity measurements (Debye 1947), a phenomenon treated initially (Kirkwood and Goldberg 1950; Stockmayer 1950) in terms of thermodynamic nonideality on the statistical-mechanical basis of excluded volume (McMillan and Mayer 1945). The absence of an analogous treatment of thermodynamic nonideality in the above section on sedimentation equilibrium reflects its recent detailed consideration elsewhere (Winzor and Wills 2025).

The nonideality parameter determined from a linear dependence of the reciprocal of light scattering intensity measurements upon solute concentration for protein solutions has routinely been identified with $\:{B}_{22}$, the osmotic second virial coefficient for solute self-interaction. However, that designation has been called into question (Deszczynski et al. 2006; Winzor et al. 2007) as the result of reports of negative values (George and Wilson 1994; Muschol and Rosenberger 1995; Rosenbaum and Zukoski 1996), a finding incompatible with interpretation of thermodynamic nonideality on the statistical-mechanical basis of excluded volume (McMillan and Mayer 1945; Hill 1968). The second virial coefficient (half of the excluded volume) for a spherical solute with radius $\:{R}_{2}$ and net charge $\:{Z}_{2}$ is given by the expressions

47a $$\:{B}_{22}=2\pi{N}_{A}\left[{\int\:}_{0}^{2{R}_{2}}{r}^{2}dr+{\int\:}_{2{R}_{2}}^{\infty}{f}_{22}\left(r\right){r}^{2}dr\right]$$

47b $$\:{f}_{22}\left(r\right)=\mathrm{e}\mathrm{x}\mathrm{p}\left[-{u}_{22}\left(r\right)/\left({k}_{B}T\right)\right]-1$$

47c $$\:\frac{{u_{{22}} \left( r \right)}}{{k_{B} T}} = \frac{{(1000Z_{2}^{{^{2} }}\kappa^{2} exp[ - (r - 2R_{2} ])}}{{(8\pi N_{A} I_{M} (1 + \kappa{R_{2}} )^{2} r)}} \:\:\:\: ;{{r \geq 2R}}_{2}$$

**Fig. 17:**
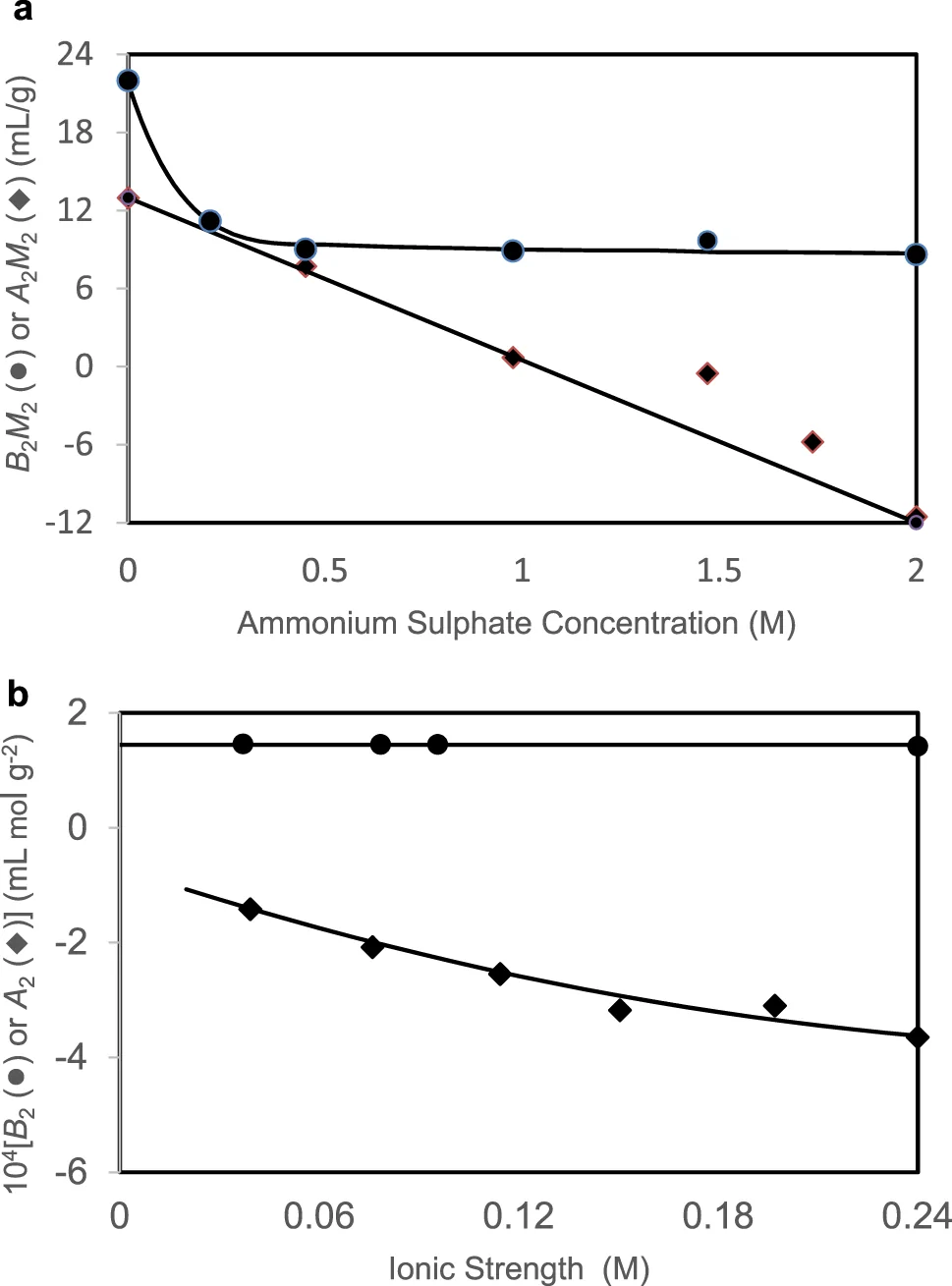
Disparity between estimates of the second virial coefficients obtained from sedimentation equilibrium ($\:{B}_{2}{M}_{2})$ and static light scattering ($\:{A}_{2}{M}_{2})\:$measurements. (**a**) Effect of ammonium sulphate concentration on $\:{B}_{2}{M}_{2}$ *(∙)* and $\:{A}_{2}{M}_{2}$ (♦) for equine serum albumin under the same conditions. [Data taken from Deszczynski et al. (2006) and Demoruelle et al. (2002) respectively. (**b**) Effect of ionic strength on $\:{B}_{2}{M}_{2}$ *(∙)*and $\:{A}_{2}{M}_{2}$ (♦) for lysozyme under similar conditions. [Dara taken from Behlke and Ristau (1999) and Muschol and Rosenberger (1995) respectively.]

The first integral in Eq. (47a) accounts for the excluded volume of two uncharged spheres (the hard sphere contribution $\:{B}_{22}^{HS})$, whereas the second integral accommodates the additional excluded volume arising from electrostatic interaction ($\:{B}_{22}^{EL}$) via the energy function $\:{u}_{22}\left(r\right)$, Eq. (47c): $\:{k}_{B}$ is the Boltzmann constant. Solution of Eq. (47a) by approximating the Mayer function, Eq. (47b), as

48 $$\:{f}_{22}\left(r\right)=\:-{u}_{22}\left(r\right)/{(k}_{B}T)+[{u}_{22}\left(r\right)/{(k}_{B}T){]}^{2}$$

leads (Winzor and Wills 2009) to the converging series shown in Eq. (49)

49 $$\:{B}_{22}=\frac{16\pi{N}_{A}{R}_{2}^{3}}{3}+ \frac{{Z_{2}^{2} (1 + 2\kappa{R_{2}} )}}{{4I_{M}(1 + \kappa{R_{2}} )^{2} }} - \frac{{(Z_{2}^{4} (1000K^{3} )(1 + 2\kappa{R_{2}} )^{2} )}}{{128\pi N_{A} I_{M}^{2} (1 + \kappa{R_{2}} )^{4} }} + \cdot\cdot\cdot$$

where the first term is the hard-sphere contribution, expressed as a molar rather than a molecular volume by the inclusion of Avogadro’s number $\:{N}_{A}$; and where the subsequent terms account for the additional excluded volume arising from charge−charge repulsion. The factor of 1000 in the final term accommodates calculation of the Debye‒Hückel inverse screening length *κ* in cm^─1^ as 3.27 ⋅ 10^7^$\:\sqrt{{I}_{M}}$ at 20 °C from the ionic strength *I* measured on the molar scale. As defined in Eq. (49) $\:{B}_{22}$ is a molar volume (mL/mol) when $\:{R}_{2}$ is measured in cm. Alternative descriptions of the excluded volume include $\:{B}_{2}={B}_{22}/{M}_{2}^{2}\:$(mL mol g^−2^) and $\:{B}_{2}{M}_{2}$ (mL/g) in experimental studies where the parameter being measured is the osmotic second virial coefficient. Because of their non-identity with osmotic counterparts the corresponding experimental parameters from light scattering measurements should be denoted as $\:{A}_{2}$ and $\:{A}_{2}{M}_{2}$. The cause for concern is first illustrated (Fig. 17a) by comparing results (Deszczynski et al. 2006) obtained by sedimentation equilibrium *(∙)* for the effect of ammonium sulphate concentration on the second virial coefficient for equine serum albumin in acetate buffer (pH 5.6) with those obtained from light scattering measurements *(⧫)*under the same conditions (Demoruelle et al. 2002). In conformity with Eq. (49), the parameter emanating from sedimentation equilibrium distributions, $\:{B}_{2}{M}_{2}$ (Wills et al. 1993) exhibits an initial decrease with increasing cosolute concentration before reaching a lower-limiting, positive asymptote that reflects the hard-sphere contribution. The corresponding dependence of the light scattering parameter ($\:{A}_{2}{M}_{2}$) upon ammonium sulphate concentration clearly bears little resemblance to that for the osmotic second virial coefficient.

Although not commented upon, evidence for the non-identity of the osmotic second virial corfficient $\:({B}_{2}{M}_{2})\:$and the corresponding light scattering parameter ($\:{A}_{2}{M}_{2}$) had already been obtained from separate studies in which light scattering (Muschol and Rosenberger 1995) and sedimentation equilibrium (Behlke and Ristau 1999) were used to examine the effect of ionic strength on the thermodynamic nonideality of lysozyme in acetate buffer, pH 4.5 (Fig. 17b). Whereas the results obtained from sedimentation equilibrium distributions *(∙)* bear a striking similarity to the present findings for equine serum albumin (Fig. 17a) in that $\:{B}_{2}{M}_{2}$ again approaches the lower-limiting positive value predicted on the statistical-mechanical basis of excluded volume (McMillan and Mayer 1945; Hill 1968), the corresponding light scattering measurements signify values of $\:{A}_{2}{M}_{2}$ that underestimate their $\:{B}_{2}{M}_{2}$ counterparts and become negative at higher ionic strengths.

We begin by attempting to seek a thermodynamic explanation for the disparate behaviour of osmotic and light scattering second virial coefficients.

### Consideration of light scattering as a thermodynamic procedure

Under the thermodynamic constraints of constant temperature and pressure (those pertaining to light scattering measurements) the chemical potential of a macromolecular solute, $\:({\mu}_{2}{)}_{T,P}$, is defined by the expression (Wills et al. 1993; Winzor et al. 2007; Winzor and Wills 2009)

50 $$\:{\left({\mu}_{2}\right)}_{T,P}=({\mu}_{2}^{o}{)}_{T,P}+RT\mathrm{l}\mathrm{n}\:{a}_{2}=({\mu}_{2}^{o}{)}_{T,P}+RT\mathrm{l}\mathrm{n}{\:(\mathrm{y}}_{2}{\mathrm{m}}_{2})$$

where the thermodynamic activity ($\:{a}_{2}$) is a molal parameter that is most simply expressed as the product of solute molality and a dimensionless molal activity coefficient $\:{y}_{2}$. As realized in the early days of light scattering (Kirkwood and Goldberg 1950; Stockmayer 1950), the thermodynamic constraint of constant pressure automatically precludes constancy of solvent chemical potential in the solutions with the different concentration $\:{c}_{2}$ that are compared experimentally. Buffer components and other small species therefore need to be considered as non-scattering cosolutes, which for the purpose of nomenclature simplification will be designated as a single cosolute (component 3) present at concentration $\:{m}_{3}{=w}_{3}/{M}_{3}$. The counterpart of the virial expansion of osmotic pressure, which is the chemical potential of solvent (species 1) under the consrtraints of constant temperature and constant solvent chemical potential, then becomes (Winzor et al. 2007; Wills et al. 2015)

51 $$\begin{aligned} \frac{{(\mu _{1} )T,P) - \mu _{1}^{o} )T,P}}{{RT}} =\frac{{w_{2} }}{{M_{2} }}+\frac{{w_{3} }}{{M_{3} }} + C_{{22}} (\frac{{w_{2} }}{{M_{2} }})^{2} + C_{{23}} \left( {\frac{{w_{2} }}{{M_{2} }}\frac{{w_{3} }}{{M_{3} }}} \right) &\\ + C_{{223}} (\frac{{w_{2} }}{{M_{2} }})^{2} \left( {\frac{{w_{3} }}{{M_{3} }}} \right) + .... \end{aligned}$$

which leads to the expression

52 $$\mathrm{ln}y_{2} = 2C_{{22}} \left( {\frac{{w_{2} }}{{M_{2} }}} \right) + C_{{23}} \left( {\frac{{w_{3} }}{{M_{3} }}} \right) + 2C_{{223}} \left( {\frac{{w_{2} }}{{M_{2} }}\frac{{w_{3} }}{{M_{3} }}} \right) + \cdot\cdot\cdot$$

for the molal activity coefficient (Hill 1968). Unlike their molar counterparts, the molal second virial coefficients $\:({C}_{22},$ $\:{C}_{23})$ and their third virial counterpart $\:{C}_{223}$ can only be expressed in terms of statistical-mechanical quantities for incompressible solutions; and in that regard assumed incompressibility is a reasonable (though imperfect) approximation for aqueous solutions. With that proviso the three parameters are interrelated by the expressions

53a $$\:{C}_{22}=\left({B}_{22}-{M}_{2}{\overline{\upsilon}}_{2}\right){\rho}_{1}$$

53b $$\:{C}_{23}=\left({B}_{23}-{M}_{2}{\overline{\upsilon}}_{2}-{M}_{3}{\overline{\upsilon}}_{3}\right){\rho}_{1}$$

53c $$\:{C}_{223}=\left({B}_{223}-2{B}_{22}{M}_{3}{\overline{\upsilon}}_{3}-2{B}_{23}{M}_{2}{\overline{\upsilon}}_{2}+2{M}_{2}{\overline{\upsilon}}_{2}{M}_{3}{\overline{\upsilon}}_{3}+{M}_{2}^{3}{\overline{\upsilon}}_{2}^{2}\right){\rho}_{1}^{2}$$

where $\:{\rho}_{1}$, the solvent density, accommodates the different dimensions of molal (mol solute per kg solvent) and molar (mol solute per litre of solution) second virial coefficients.

For a single solute the concentration dependence of the Rayleigh excess scattering ratio $\:{R}_{\theta}$. is described by the relationship (Debye 1947)

54 $$\:\frac{{K}_{s}{c}_{2}}{{R_\theta}_{}}=\frac{1}{{M}_{2}} ~2A_{2} c_{2} + \cdot\cdot\cdot$$

where $\:{K}_{s}$ is an optical constant, and where the light scattering second virial coefficient,

55 $$A_2=\:\frac{{C}_{22}/{\rho}_{1}+{M}_{2}{\overline{\upsilon}}_{2}-{M}_{2}(dn/d{c}_{2})/{n}_{1}}{{M}_{2}^{2}}= \frac{{(B_{{22}} - M_{2} (dn/dc_{2} )/n_{1} )}}{{(M_{2}^{2} )}}$$

is essentially the osmotic second virial coefficient $\:{B}_{22}$, but decreased slightly by a refracrive index term in which $\:{n}_{1}$ denotes the solvent refractive index: the specific refractive index increment (dn/d$\:{c}_{2})$ will henceforth be designated as $\:{\chi}_{2}$. The error resulting from neglect of this term would only lead to overestimation of $\:{B}_{22}$ by about 2.5% for isoelectric ovalbumin ($\:{M}_{2}$ = 44 kDa), which is probably within the experimental uncertainty of $\:{A}_{2}$ measurements.

The presence of a small nonscattering cosolute at concentration $\:{c}_{3}$ introduces greater complexity into the Debye equation, which becomes (Winzor et al. 2007)

56a $$\:\frac{{K}_{s}{c}_{2}}{{R}_{\theta}}=\frac{{A}_{1}}{{M}_{2}}\:+\:2{A}_{2}{c}_{2}+\:\dots\:\:$$

56b $$\:{A}_{1}\:=\:1+2\left({ \chi }_{3}/{ \chi }_{2}\right)\left[{C}_{23}/\left({M}_{2}{\rho}_{1}\right)+{\overline{\upsilon}}_{3}\right]{c}_{3}\:$$

56c $$\:{A}_{2}\:=\:\frac{{C}_{22}+{M}_{2}{\overline{\upsilon}}_{2}-{M}_{2}{ \chi }_{2}/{n}_{s}+ \Omega {c}_{3}/2}{{M}_{2}^{2}{M}_{3}}\:$$

56d $$\begin{aligned} \Omega = \:[C_{{223}} /\rho _{1}^{2} - \:(C_{{23}} /\rho _{1} )^{2} + 2(C_{{22}} - C_{{23}} )M_{3} \bar{v}_{3} /\rho _{1}\\ + 4C_{{22}} M_{3} \left( {\chi _{3} /\chi _{2} } \right)\bar{v}_{2} /_{1} \: + 2\{ (\chi _{3} M_{{3)}} /(\chi _{2} M_{2} )\} \\\{ C_{{223}} /\rho _{1}^{2} + 2C_{{22}} C_{{23}} /\rho _{1}^{2} + 2(C_{{23}} M_{2} /\rho _{1} )[\bar{v}_{2} - (\chi _{2} /n_{s} )]\} \: \\+ M_{2} [6(\chi _{3} /\chi _{2} )\bar{v}_{2}^{2} - 4(\chi _{3} /n_{s} )\bar{v}_{2} ] \\ \end{aligned}$$

after incorporating the experimental convention that the optical constant is based on the buffer refractive index $\:{n}_{s}\:$rather than that, $\:{n}_{1}\:$of solvent (water).

**Fig. 18:**
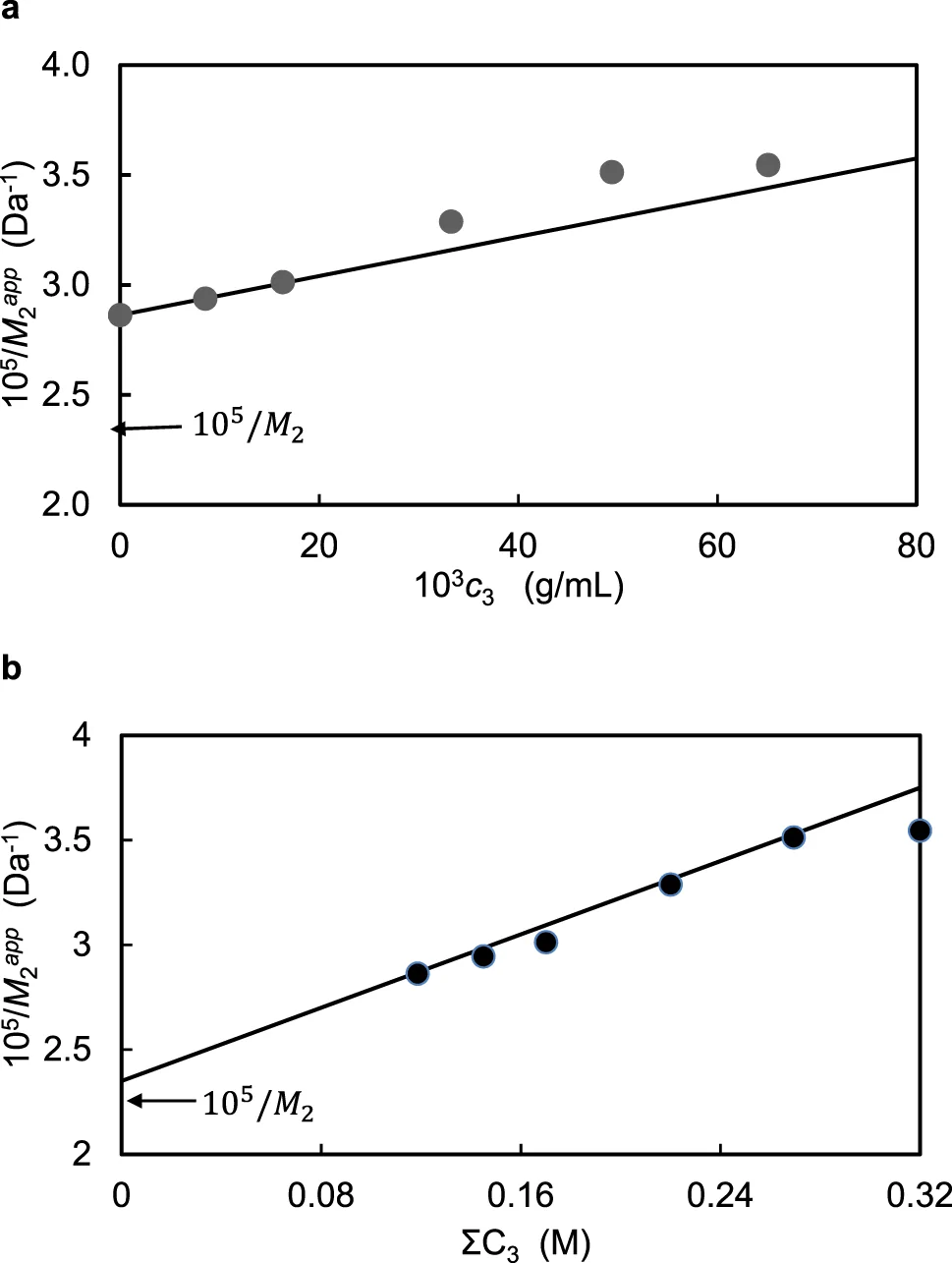
Experimental verification that the ordinate intercept of a Debye plot deviates from $\:1/{M}_{2}$ in cosolute-suppemented solvent. (**a**) Comparison of the effect of sucrose concentration ($\:{c}_{3}$) on the ordinate intercept ($\:1/{M}_{2}^{app}$) for isoelectric ovalbumin with the dependence predicted by Eq. (56b). (**b**) Corresponding effect on $\:1/{M}_{2}^{app}$ for isoelectric ovalbumin with the small-solute concentration (Σ$\:{C}_{3})$ supplemented by including the concentrations (0.04 M) of buffer species (acetic acid as well as sodium and acetate ions) buffer. [Data in (**a**) and (**b**) taken from Fig. 4 of Winzor et al. (2007).]

The first point to note from this thermodynamic consideration of cosolute effects on light scattering measurements is the prediction [Eq. (56a)] that the ordinate intercept of the Debye plot is no longer $\:1/{M}_{2}$. Such linear dependence of the ordinate intercept was demonstrated for non-aqueous polymer solutions in the middle of last century (Kirkwood and Goldberg 1950) but has only resurfaced (for aqueous protein solutions) in the past two decades (Winzor et al. 2007; Blanco et al. 2012). This behaviour is illustrated in Fig. 18a, which summarizes the effect of sucrose supplementation on the ordinate intercept of the Debye plot *(∙)* for isoelectric ovalbumin (pH 4.6, I 0.04 M). As noted in the report of those studies (Winzor et al. 2007), conformity with predicted behaviour is observed in the sense that the theoretical slope (^_____^) calculated from Eq. (56b) affords an acceptable description of those experimental results. Furthermore, noncompliance with the predicted ordinate intercept ($\:1/{M}_{2}$) was attributed to neglect of the consequences of non-scattering buffer components, to which excluded-volume theory is inapplicable. Circumstantial evidence in support of that contention (Scott et al. 2013) comes from the ionic strength dependence of $\:1/{M}_{2}^{app}$ for isoelectric ovalbumin reported in Fig. 4 of the above study (Winzor et al. 2007). That approach is now shown in Fig. 18b, where we re-analyse the results presented in Fig. 18a with the small-solute concentration (molar) supplemented by the inclusion of those (0.04 M) for buffer species (acetic acid as well as sodium and acetate ions ‒ an approximation that should suffice for illustrative purposes). Linear extrapolation of those results (*∙*, Fig. 18b) certainly yields an ordinate intercept that is a better estimate of $\:1/{M}_{2}$for this glycoprotein with a calculated molecular mass of 44 kDa (Hall et al. 1999). 

The second point to note in relation to Eq. (56c) is the presence of a negative $\:({C}_{23}/{\rho}_{1}{)}^{2}$ term in the expression for *Ω*, Eq. (56d). However, this negative contribution is effectively offset by the positive counterpart emanating from the $\:{C}_{223}/{\rho}_{1}^{2}$ term, which can be calculated from Eq. (53c) on the basis (Blaak 1998) that

57 $$\:{B}_{223}=[16{\pi}^{2}{N}_{A}^{2}/9]\:[{R}_{2}^{6}+2{R}_{2}^{3}{R}_{3}^{4}+3{R}_{2}^{2}{R}_{3}\left(2{R}_{2}+{R}_{3}\right)({R}_{2}^{2}+2{R}_{2}{R}_{3}\left)\right]$$

**Fig. 19:**
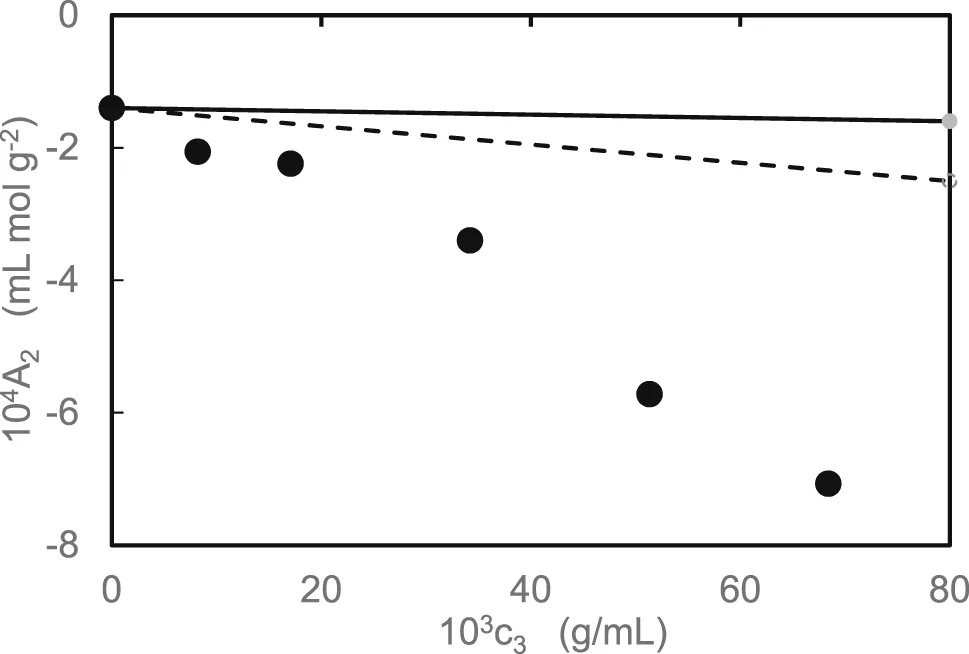
Effect of buffer supplementation with sucrose on the measured light scattering second virial coefficient *(∙)* for isoelectric ovalbumin (pH 4.6, $\:{I}_{M}$ 0.04). The broken line describes the theoretical dependence (Winzor et al. 2007) obtained from Eq. (56c) by neglecting the $\:{C}_{223}/{\rho}_{1}^{2}$ term; and the solid line the revised theoretical dependence after allowance for the third virial coefficient contribution via Eqs. (53c) and (57). [Data taken from Fig. 5 of Winzor et al. (2007).]

Although the predicted Ω$\:{c}_{3}$ contribution to the light scattering second virial coefficient is slightly negative for isoelectric ovalbumin in the presence of sucrose, it is considerably smaller than that reported earlier by neglecting the $\:{C}_{223}$term (Fig. 19). Present considerations thus reinforce the earlier conclusion (Winzor et al. 2007) that the experimentally observed dependence of $\:{A}_{2}$ upon sucrose concentration is not amenable to statistical thermodynamic explanation in terms of excluded volume. An additional source of negative contributions to the light scattering second virial coefficient must therefore be sought to achieve its description solely on thermodynamic grounds.

Inasmuch as any chemical interaction within the thermodynamic system under consideration leads to a decrease in $\:{A}_{2}$, the negative light scattering second virial coefficients reported for protein solutions supplemented with high concentrations of small cosolutes could well include additional negative contributions from protein−cosolute, solute−solvent and cosolute−solvent interactions. In that regard the existence of anion and cation binding to proteins is well established in some situations (Scatchard et al. 1950; Carr 1953,1956). Furthermore, the hydrogen-bonding ability of water to change its own structural state has implications for its potential interaction with polyol cosolutes such as saccharides as well as the oxygen atom in the polyethylene glycol repeat unit. As pointed out in a recent reappraisal of isopiestic measurements on aqueous urea solutions (Winzor and Wills 2019), solute−solute, solute−solvent and solvent−solvent hydrogen bonding interactions all contribute to the thermodynamics of urea dimerization.

#### Consideration of $\:{\boldsymbol{A}}_{2}\:$as a steady-state hydrodynamic parameter

A lack of success in the above attempt to obtain a satisfactory quantitative description of $\:{A}_{2}$ prompted a switch to the possibility that static light scattering measurements $\:({R}_{\theta})$ may reflect the attainment of a hydrodynamic steady state rather than thermodynamic equilibrium (Winzor et al. 2023). In view of the insensitivity of predicted $\:{A}_{2}{M}_{2}$ values to concentration of buffer/added cosolutes as additional components (Fig. 19), we proceed on the basis that nonideality in light scattering measurements on buffered protein solutions can be described adequately by single‒solute theory with $\:{C}_{22}/{\rho}_{b}$ taken as $\:{B}_{22}-{M}_{2}{\overline{\upsilon}}_{2}$ on the grounds that $\:{{\rho}_{b}}\approx\:1$ g/mL for aqueous buffers.

The first detailed treatment of hydrodynamic interactions in Brownian motion coupled with the net flux of solute was provided by Batchelor (1976) in the context of concentration-dependent diffusion in sedimentation velocity for sufficiently dilute solutions to justify the restriction of nonideality considerations to pairwise interactions between particles. In that theoretical study a combination of statistical-mechanical and hydrodynamic approaches led to description of the concentration dependence of the diffusion coefficient *D* for a rigid uncharged spherical particle under those dilute conditions as

58a $$D = \frac{{[1 + (8\phi - 6.55\phi )](k_{B} T)}}{{6\pi n_{1} R_{2} }} = D_{o} [1 + (k_{T} - k_{H} )\phi = D_{o} (1 + k_{D}^{{SM}} C_{2} )$$

in which Φ, the volume fraction occupied by the diffusing particle, is the product of the weight-concentration $\:{c}_{2}$ (g/mL) of solute and its solvated specific volume $\:{v}_{s}$,

58b $$\:{v}_{s}\:=\:\frac{4\pi{N}_{A}{R}_{2}^{3}}{\left(3{M}_{2}\right)}\:$$

and $\:{D}_{o}$ is the translational diffusion coefficient obtained experimentally in the limit of zero solute concentration:

58c $$\:{D}_{o}\:=\:\frac{{k}_{B}T}{6{\pi}\eta_{1}{R}_{2}}\:$$

In that regard the concentration coefficient has been designated as $\:{k}_{D}^{SM}$ to signify that only statistical-mechanical factors have been considered.

From Eq. (49) it is evident that the factor $\:{k}_{T}=8\phi$ in Eq. (58a) corresponds to $\:2{B}_{22}/{M}_{2}$, the volume from which the centres of two uncharged spherical solute molecules are mutually excluded; and is therefore a thermodynamic factor. Hydrodynamic factors are incorporated into the second term $\:{k}_{H}=6.55\phi$, which decreases the effective magnitude of the excluded volume. On the grounds that the osmotic second virial coefficient equates with half of the thermodynamic contribution, the same situation applies to its hydrodynamic counterpart. The excluded volume contribution to the concentration coefficient $\:{k}_{D}^{SM}$ in Eq. (58a) thus becomes $\:\left[\left({k}_{T}-{k}_{H}\right){v}_{s}\right]/2$ to be consistent with the description of $\:{k}_{T}$ as $\:2{B}_{22}^{HS}$/$\:{M}_{2}$.

Subsequent attention has been directed towards the determination of *D* from dynamic light scattering studies, where the same expression, Eq. (58a), has also been obtained for an uncharged spherical particle (Felderhof 1978; Wills 1979; Phillies and Wills 1981; Cichocki and Felderhof 1988). As noted by Petsev and Denkov (1992), the presence of net charge on those hard spheres increases the magnitude of the thermodynamic term in accordance with Eqs. (47a‒c). They also showed that the corresponding terms for the hydrodynamic contribution are dominated by the Oseen contribution ($\:{\wedge}_{O}{v}_{s}$)

59 $$\wedge _{O} v_{s} = \frac{{(2\pi N_{A} R_{2} )}}{{M_{2} }}[\int_{0}^{{2R_{2} }} {rdr} + \int_{{2R_{2} }}^{\infty } {f_{{22}} } (r)]$$

which establishes that the Oseen hard-sphere contribution to $\:{k}_{H}$ is 6*φ*: the remainder (0.55*Φ*) comes from two other contributions that account for short-range hydrodynamic interactions (Felderhof 1978; Petsev and Denkov 1992).

The coefficient describing the concentration dependence of the statistical mechanical contribution to the diffusion coefficient, $\:{k}_{D}^{SM}=({k}_{T}-{k}_{H}){v}_{s}/2$, was therefore considered to be given by the expression (Winzor et al. 2023)

60 $$k_{D}^{{SM}} = {\frac{{(k_{T}^{{HS}} - k_{H}^{{HS}})\upsilon_S }}{2}} + \frac{{2\pi N_{A} }}{{M_{2} }}\int_{{2R_{2} }}^{\infty } {f_{{22}} } (r)r^{2} dr + \frac{{2\pi N_{A} R_{2} }}{{M_{2} }}\int_{{2R_{2} }}^{\infty } {f_{{22}} } (r)rdr$$

Although the two integrals in Eq. (60) are usually evaluated by means of an approximate expression for $\:{f}_{22}\left(r\right)$, Eq. (48), trapezoidal integration in accordance with Eqs. (47a‒c) was used (Winzor et al. 2023) to avoid the risk of $\:{k}_{T}{v}_{s}$ (and presumably $\:{k}_{H}{v}_{s}$) overestimation (Winzor and Wills 2009).

In dynamic light scattering studies ($\:{k}_{T}^{HS}-{k}_{T}^{HS})$ has usually been taken as 1.45 for dilute solutions of rigid spherical particles, which was the value deduced by Batchelor (1976) for diffusion effected by a concentration gradient in sedimentation velocity measurements. The use of a lower value, ‒0.9, has been recommended by Phillies (1987) on the grounds that light scattering spectroscopy is merely sensitive to particle position: that stance is adopted here.

### Comparison of static and dynamic light scattering measurements

**Fig. 20:**
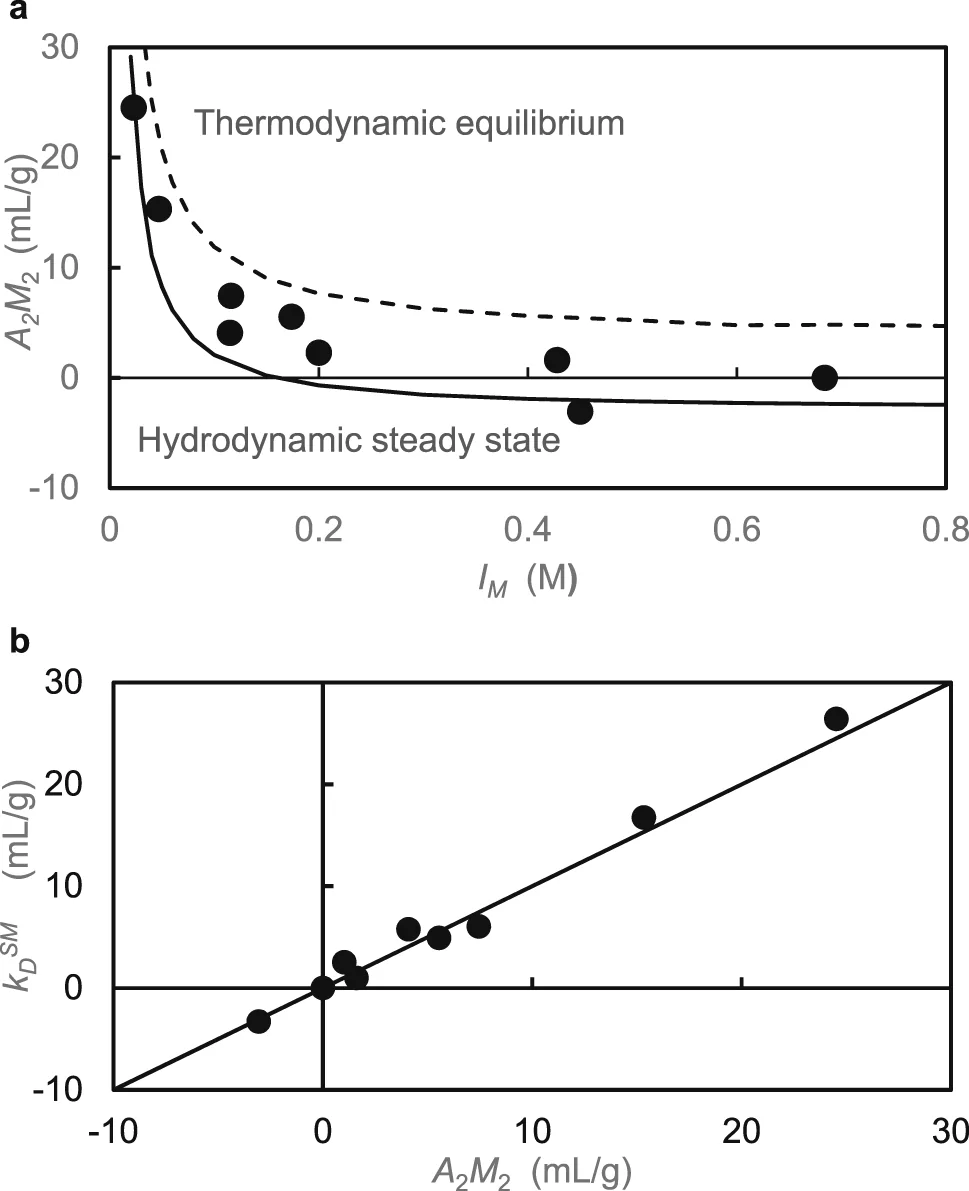
Analysis of published static light scattering data (Muschol and Rosenberger 1995) for lysozyme solutions at pH 4.7. (**a**) Experimental results *(∙)* for the dependence of the second virial coefficient upon ionic strength, together with theoretical dependence predicted either on the basis of its consideration as a thermodynamic parameter,$\:\:{A}_{2}{M}_{2}=\:{k}_{T}{v}_{s}/2-{\stackrel{-}{v}}_{2}$, (‒ ‒ ‒), or the combination of that parameter and its statistical-mechanical hydrodynamic counterpart, $\:{k}_{D}^{SM}$ (^______^). (**b**) Demonstration of the correlation between $\:{k}_{D}^{SM}\:$and $\:{A}_{2}{M}_{2}$ [Data taken from Fig. 2 of Winzor et al. (2023).]

Ionic strength dependence of the light scattering second virial coefficient for lysozyme in acetate‒chloride buffers, pH 4.7, I 0.02‒0.80) is shown in Fig. 20a, where experimental points *(∙)* refer to values of $\:{A}_{2}$ reported in Table 2 of Muschol and Rosenberger (1995) based on a molar mass of 14.6 kDa. Attempts (Winzor et al. 2023) to describe those results in thermodynamic terms, $\:{A}_{2}{M}_{2}=\:{k}_{T}{v}_{s}/2-{\stackrel{-}{v}}_{2},$ with respective values of 1.9 nm and 11 for $\:{R}_{2}$ and $\:{Z}_{2}$ ((Muschol and Rosenberger 1995) to solve the integrals in Eq. (47a‒c) clearly led to their consistent overestimation (‒ ‒ ‒), thereby corroborating earlier assertions that the light scattering coefficient should not be regarded as the osmotic second virial coefficient (Deszczynski et al. 2006; Winzor et al. 2007; Wills et al. 2015).

Much better agreement between experiment and prediction was achieved (^_____^, Fig. 20a) by regarding the light scattering second virial coefficient $\:{A}_{2}{M}_{2}$ as a monitor of $\:\left[({k}_{T}-{k}_{H}){v}_{s}/2\right]$, which identifies it as a hydrodynamic rather than a thermodynamic parameter on the grounds, Eq. (60), that $\:{A}_{2}{M}_{2}$ equates with $\:{k}_{D}^{SM}$, the concentration dependence coefficient governing the statistical-mechanical contribution to translational diffusion in a Brownian system. However, account needs to be taken of the effects of viscosity on the magnitude of the measured diffusion coefficient at finite protein concentrations before direct experimental testing of that premise. This deficiency is remedied by re-writing the final version of Eq. (58a) as (Scott et al. 2014)

61 $$\:D=\:\frac{{D}_{o}(1+{k}_{D}{c}_{2})\:}{\eta/{\eta}_{b}}$$

where *η* denotes the viscosity of a protein solution with concentration $\:{c}_{2}$ for which *D* was measured and ${\eta}_{b}$ that of buffer (the solution viscosity in the limit of zero concentration to which $\:{D}_{o}$ refers). Allowance for the fact that the relative viscosity is related to the intrinsic viscosity [*η*] of a spherical protein species by the expression (Tanford 1961)

62 $$\eta/\eta_{b} = 1 + [\eta]c_{2} + ... \approx 1 + 2.5v_{s} c_{2}$$

decreases the experimental value of $\:{k}_{D}$ by 2.5$\:{v}_{s}$. A zero ordinate intercept and a slope of unity is thus the predicted dependence of $\:{k}_{D}^{SM}={(k}_{D}+2.5{v}_{s})$ upon $\:{A}_{2}{M}_{2}$ for a system in which $\:{R}_{\theta}$ reflects the attainment of a hydrodynamic steady state. Such behaviour is evident in Fig. 20b, where the values of $\:{k}_{D}$ for lysozyme have been inferred (Winzor et al. 2023) from Table 2 of Muschol and Rosenberger (1995). Further support is thereby provided for identification of the light scattering second virial coefficient with the statistical mechanical contribution to $\:{k}_{D}$, [($\:({k}_{T}-{k}_{H}){v}_{s}/2]-{\stackrel{-}{v}}_{2}$, which was the conclusion drawn from Fig. 20a.

**Fig. 21:**
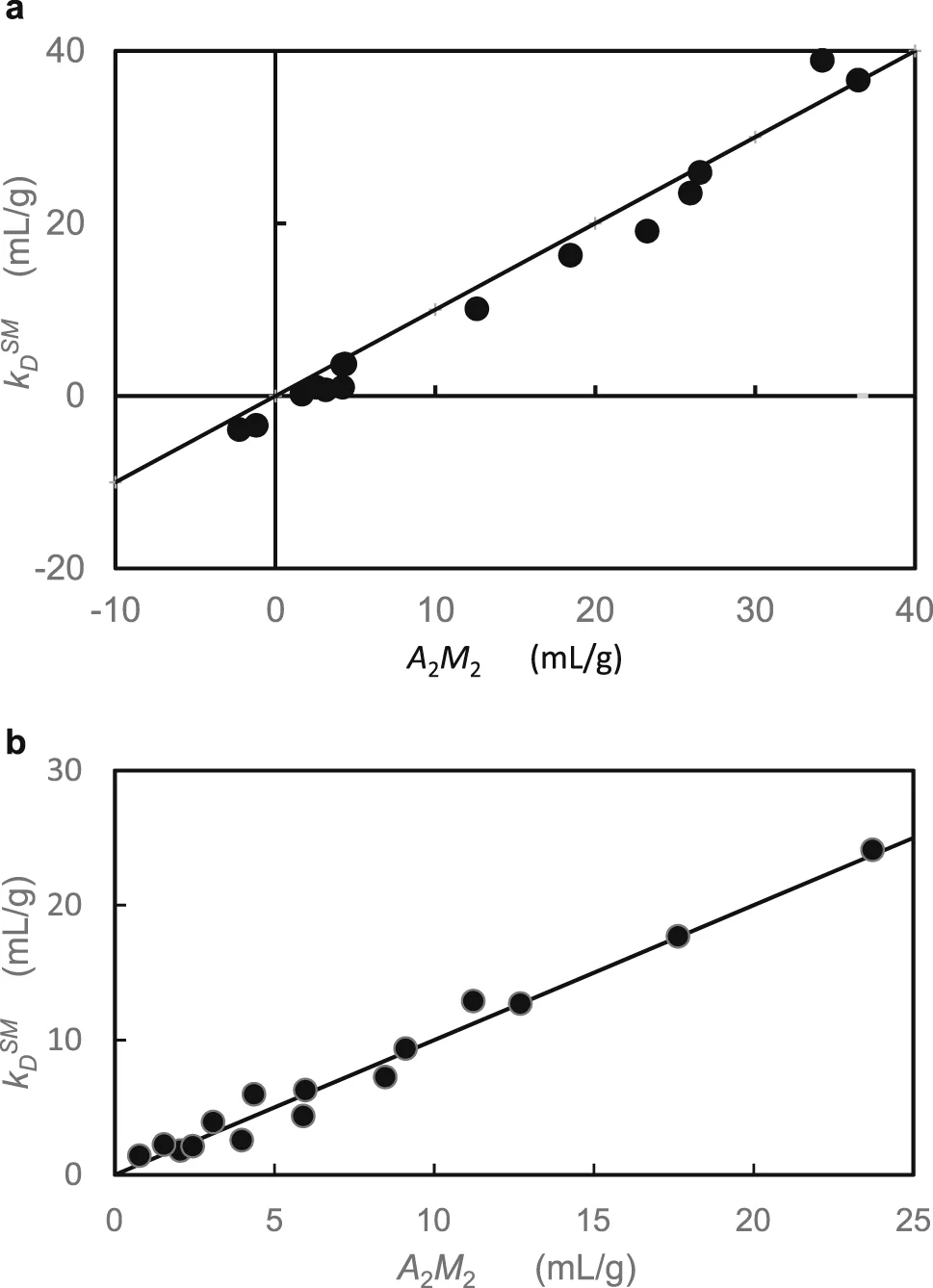
Further evidence of correlation between the statistical-mechanical contribution to the diffusion concentration coefficient $\:({k}_{D}^{SM}$) and the light scattering second virial coefficient $\:{(A}_{2}{M}_{2}$) for monoclonal IgG antibodies. (**a**) Combined results for five monoclonal antibodies (pH 6.0) at a range of ionic strengths. [Data inferred from Lehermayr et al. (2011).] (**b**) Corresponding dependence for a single monoclonal antibody at pH 5.0 and pH 5.75 at a range of ionic strengths. [Data inferred from Roberts et al. (2014).]

As noted previously (Winzor et al. 2023), results analogous to those for lysozyme (Fig. 20b) have been reported for monoclonal antibodies (Lehermayr et al. 2011; Roberts et al. 2014). In the current plots of those data (Fig. 21a and b) only the statistical-mechanical contribution has again been used as the ordinate parameter to emphasize the essential identity of the magnitudes of the statistical mechanical contribution to $\:{k}_{D}$ and the light scattering second virial coefficient.

The results presented in Figs. 20 and 21 seemingly provide a logical explanation for the failure to obtain a satisfactory thermodynamic description of the time-independent $\:{R}_{\theta}$ values observed in static light scattering experiments. Although those results are amenable to statistical-mechanical interpretation, that description involves a hydrodynamic ($\:{k}_{H}$) as well as a thermodynamic ($\:{k}_{T}$) contribution. Despite the suggestion of that possibility many years ago (Neal et al. 1990), the consideration of $\:{R}_{\theta}$ as an equilibrium parameter persisted for another two decades. The above evidence (Winzor et al. 2023) clearly questions the current thermodynamic status accorded static light scattering. Indeed, the need for its reclassification as a hydrodynamic steady-state procedure is indicated.

### Concluding remarks

From the viewpoint of ascertaining whether the observation of a time-independent signal reflects the attainment of thermodynamic equilibrium or merely a kinetic (or hydrodynamic) steady state, analytical ultracentrifugation is a single technique that provides a wide range of examples. In sedimentation velocity studies of a single non-associating solute the diffusional spreading of boundaries is always countered by negative concentration dependence of the sedimentation coefficient, but for globular proteins the extent of this boundary sharpening does not suffice to generate a hydrodynamic steady state (Fig. 1). On the other hand, the greater extent of *s*‒*c* dependence exhibited by flexible macromolecules affords an opportunity for spontaneous generation of a steady state (Fig. 2).

Early analyses of sedimentation equilibrium distributions were based on their description in terms of a hydrodynamic steady state across the entire liquid column being centrifuged [Eqs. (4) and (15)]. Although this approach found limited use for protein solutions because of the length of time (weeks) required to attain that steady state across a 0.9 cm column, its valid application to the situation pertaining at either extreme of the liquid column (the air‒liquid meniscus and the cell base) provided a major breakthrough in the use of sedimentation equilibrium for molecular weight measurement (Fig. 4).The reign of this “approach-to-equilibrium” version of the technique ended abruptly with the realization that sedimentation equilibrium attainment throughout the liquid column could be achieved within 24 h by a 3- to 5-fold decrease in its length ‒ a development prompted by the investigation (Goldberg 1953) that accorded full thermodynamic status to the procedure.

Studies of protein‒ligand interactions by migration methods such as sedimentation velocity, moving boundary electrophoresis and chromatography provide numerous examples of situations involving the establishment of kinetic steady states. Fortunately, this state of affairs in quantitative studies of protein‒ligand mixtures by moving boundary electrophoresis was recognized and accommodated at the outset by combining the necessity for mass conservation with the concept of constituent quantities (Fig. 6). Only in the event that the acceptor and complex(es) comigrate ($\:{v}_{C}={v}_{A})$ can the concentration of free ligand in the trailing boundary of a descending pattern ($\:{C}_{B}^{\beta}$) be accorded thermodynamic status ($\:{C}_{B}^{\beta}={C}_{B}^{\alpha}).$ Steinberg and Schachman (1966) took advantage of this simplification in sedimentation velocity studies of albumin‒methyl orange mixtures (Fig. 8). On the other hand, adoption of the same approach in studies of interactions between small ligands (proflavine, aminoacridines) and nucleic acid by sedimentation velocity (Lloyd et al. 1968; Blake and Peacocke 1968) was demonstrably invalid because of the non-identity of $\:{s}_{A}$ and $\:{s}_{C}$. Misidentification of the free ligand boundary as the thermodynamic concentration $\:{C}_{B}^{\alpha}$ also occurred in studies of protein‒poly(styrenesulphonate) mixtures by frontal analysis continuous capillary electrophoresis (Fig. 7).

Despite the obvious loss of a region with the original composition in zonal studies of protein‒ligand interactions, quantitative characterization can still be achieved by subjecting a small zone of acceptor solution to chromatography or electrophoresis in a medium with defined ligand concentration (Fig. 12a and b). However, experimental verification of the identity of migration rates, elution volumes or retention times for protein and protein‒ligand complex(es) is a prerequisite for validity of the approach. This requirement was met in the original application of this Hummel‒Dreyer (1962) approach by employing a size-exclusion matrix (Sephadex G50) that excluded the protein and hence protein‒ligand complex(es) from the stationary phase. An attempt to characterize ligand interaction with a small acceptor by the same approach (Colman 1972) was invalidated by the establishment of a kinetic steady state rather than thermodynamic equilibrium [Eq. (54)‒(56); Fig. 13]. The same problem can arise in techniques such as affinity capillary electrophoresis, where the major uncertainty in the magnitude of the evaluated binding constant stems from consideration of the measured retention time to reflect migration in the medium with ligand concentration $\:{(C}_{B}{)}_{p}$ instead of the mean of steady-state concentrations across the migrating zone, ($\:{C}_{B}{)}_{av}$. However, as noted above, this substitution can be a reasonable approximation in instances where the acceptor concentration in the migrating zone ($\:{\overline{C}}_{A}$) is much smaller than $\:{(C}_{B}{)}_{p}\:$[see Eqs. (40) and (41) as well as Figs. 12b and 13].

A need for distinction between attainment of a kinetic steady state and thermodynamic equilibrium also occurs in quantitative studies of the binding of a macromolecular ligand (B) to a highly concentrated array of small solute acceptors (A) on a polymer lattice. This situation first came to light (Latt and Sober 1967; McGhee and von Hippel 1974; Greene and Eisenberg 1980; Ols0n et al. 1991; Munro et al. 1998,2000) in spectrophotometric studies of 1:1 interaction between proteins and polymer chains comprising a linear array of identical or very similar repeat units (acceptor sites A). Although any residue sequence of appropriate length (*n*) is a potential acceptor site, the attachment of a large ligand to one such unit also entails the coverage (masking) of a larger length of residue sequence (*m*). Random attachment of protein can thus lead to suboptimal alignment of ligand molecules along the polymer chain.

A parking problem (Hill et al. 1980; Greene and Eisenberg 1980) is thereby generated whereby additional dissociation and reassociation events are required to convert a time-independent ligand-binding response reflecting attainment of a kinetic steady-state to one reflecting the establishment of thermodynamic equilibrium (Fig. 15a, b). Even then the binding curve exhibits a form generally ascribed to negative cooperativity of acceptor sites; but in these circumstances the deviation from rectangular hyperbolic binding behaviour arises because the concentration of available acceptor sites for successive ligand attachment, $\:({\overline{C}}_{A}-m{C}_{C}$), decreases more rapidly than the corresponding number, $\:({\overline{C}}_{A}-{C}_{C})$, commensurate with equivalence and independence of univalent ligand binding. An experimental example of this phenomenon is illustrated with the binding of thrombin to heparin (Fig. 16).

This review ends on a controversial note by questioning the thermodynamic status of static light scattering measurements. From the outset concentration dependence of the time-independent signal being monitored (turbidity *τ* initially but replaced by the Rayleigh excess ratio $\:{R}_{\theta}$) was based on the premise of thermodynamic equilibrium attainment to obtain the solute molecular weight $\:{M}_{2}$ and a second virial coefficient $\:{A}_{2}{M}_{2}$ via the Dehye equation. For years the latter parameter was identified with the corresponding osmotic second virial coefficient $\:{B}_{2}{M}_{2}$ for a single-solute system even though the operative thermodynamic constraints (constant *T*, *P*) dictated the need to regard buffer components in protein solutions as small non-scattering cosolutes (Kirkwood and Goldberg 1950; Stockmayer 1950). Experimental evidence refuting the identity of these two parameters only surfaced much later (Deszczynski et al. 2006; Winzor et al. 2007; Behlke and Ristau 1999) in response to reports of negative osmotic second virial coefficients for protein self-interaction obtained by static light scattering (George and Wilson 1994; Muschol and Rosenberger 1995; Rosenbaum and Zukoski 1996): $\:{B}_{2}{M}_{2}$ is a thermodynamic parameter that is necessarily positive on the statistical-mechanical basis of excluded volume (McMillan and Mayer 1945; Hill 1968). Failure of attempts to describe the experimental variation of $\:{A}_{2}{M}_{2}$ by incorporation of thermodynamic excluded volume contributions arising from protein‒cosolute interactions (Winzor et al. 2007) has finally led to the demonstration (Winzor et al. 2023) that better agreement between experiment and theoretical prediction is obtained by identifying $\:{A}_{2}{M}_{2}$ with the statistical-mechanical contribution $\:{k}_{D}^{SM}$ to the coefficient for concentration dependence of the translational diffusion coefficient. Because that treatment of $\:{k}_{D}$ includes statistical-mechanical contributions to nonideality arising from hydrodynamic interactions (Batchelor 1976), $\:{A}_{2}{M}_{2}$ ceases to be a thermodynamic parameter. In other words, the time-independence of the excess Rayleigh ratio reflects the attainment of a hydrodynamic steady state, not thermodynamic equilibrium.

In conclusion, the need for distinction between attainment of a kinetic steady state rather than thermodynamic equilibrium as the source of time-independent experimental responses extends across a wide range of biological disciplines and techniques for the quantitative characterization of macromolecular interactions. In studies of protein interactions by sedimentation equilibrium the time-independent response can be regarded as either a kinetic steady state (Lamm 1929) or a system in thermodynamic equilibrium (Goldberg 1953). The kinetic steady state also reflects the attainment of thermodynamic equilibrium in size-exclusion chromatographic studies of protein‒ligand interactions under conditions where the protein and protein‒ligand complex(es) comigrate (Hummel and Dreyer 1962; Nichol and Winzor 1964; Cooper and Wood 1968; Nichol et al. 1971, 1972), but not when that restrictive condition fails to apply (Colman 1972; Cann and Winzor 1987; Cann et al. 1989). This review serves to emphasize that reliable quantitative characterization of macromolecular interactions from experimental responses reflecting only kinetic steady state attainment is still possible once the deviation from thermodynamic equilibrium is recognized.
